# From Brewing By-Products to Next-Generation Food Ingredients: Processing, Functionality, Safety, and Industrial Translation

**DOI:** 10.3390/foods15122193

**Published:** 2026-06-17

**Authors:** Ionut-Dumitru Veleșcu, Ioana Cristina Crivei, Andreea Bianca Balint, Florina Stoica, Florin Daniel Lipșa, Roxana Nicoleta Rațu

**Affiliations:** 1 Faculty of Agriculture, Department of Food Technologies, “Ion Ionescu de la Brad” Iasi University of Life Sciences, 3 Mihail Sadoveanu Alley, 700489 Iasi, Romania; ionut.velescu@iuls.ro (I.-D.V.); bianca.balint@iuls.ro (A.B.B.); florin.lipsa@iuls.ro (F.D.L.); 2 Faculty of Agriculture, Department of Pedotechnics, “Ion Ionescu de la Brad” Iasi University of Life Sciences, 3 Mihail Sadoveanu Alley, 700489 Iasi, Romania; florina.stoica@iuls.ro

**Keywords:** food ingredients, upcycled food, techno-functional properties, food safety, brewing by-products

## Abstract

Brewing generates several by-products with high potential for conversion into food in-gredients, including brewer’s spent grain, brewer’s spent yeast, spent hops, and hot trub. These streams contain dietary fibre, proteins, β-glucans, phenolics, minerals, and others with nutritional and technological value. This review evaluates their suitability for food applications by linking composition, processing routes, techno-functional behaviour, safety, sensory quality, and industrial readiness. A structured literature search covering publications from 2015 to 2026 was conducted in Web of Science, Scopus, PubMed, and Google Scholar to support a critical narrative synthesis of food-relevant applications of brewing by-products. The review shows that brewer’s spent grain is the most suitable by-product for wider food use, mainly in bakery, snacks, pasta, and cereal-based products, due to its high availability and fibre-rich composition. Brewer’s spent yeast is more appropriate for fraction-based applications involving proteins, peptides, β-glucans, and mannoproteins, especially in dairy products, savoury foods, beverages, and encapsula-tion systems. Spent hops and hot trub are less suitable for direct incorporation, but they may be used for selective recovery of phenolic-rich, antioxidant, flavour-active, or pro-tein-containing fractions. The conversion of these materials into food ingredients depends strongly on stabilization, drying, milling, extraction, fermentation, enzymatic treatment, debittering, and fractionation. Main limitations include high moisture content, short shelf-life, microbial spoilage, compositional variability, bitterness, dark colour, high nucleic acid content in yeast-derived fractions, regulatory uncertainty, and limited pilot-scale validation. Overall, brewing by-products can support the development of up-cycled ingredients when processing, safety, sensory quality, and product compatibility are controlled. Future progress requires standardized recovery protocols, stronger quality control, sensory validation, legal assessment, and scale-up studies to support their use in commercial food production.

## 1. Introduction

Beer is one of the oldest fermented beverages and remains an important product of the global food and beverage sector, supported by a complex value chain that includes agriculture, malting, brewing, packaging, distribution, and retail. Large-scale beer production generates economic value and employment, but it also produces substantial amounts of solid and liquid by-products that require appropriate management to avoid environmental, logistical, and resource-efficiency challenges [1,2,3].

The brewing process is based on the fermentation of starch-rich raw materials by yeasts, mainly *Saccharomyces pastorianus* in lager production and *Saccharomyces cerevisiae* in ale production, although other yeast species may also contribute to aroma formation and process diversification [4]. Malt, hops, water, and adjunct grains are the main raw materials used, depending on beer style, formulation, and production scale. In addition to beer, the process generates several residual streams with distinct physicochemical characteristics, compositional profiles, and recovery potential [1,5].

From an industrial and environmental perspective, the most relevant brewing by-products include brewer’s spent grain (BSG), brewer’s spent yeast (BSY), spent hops (BSH), hot trub, and brewery process effluents. These streams are generated continuously and in large volumes, and their accumulation can create environmental and economic burdens when storage, transport, stabilization, or disposal are not properly managed [6,7,8]. Fresh brewing residues are particularly challenging because of their high moisture content, variable composition, and susceptibility to rapid microbial deterioration, all of which restrict direct reuse and may promote low-value disposal routes [3,9,10].

However, these streams should not be regarded only as wastes. Brewing by-products contain valuable compounds, including dietary fibre, proteins, peptides, β-glucans, mannoproteins, phenolic compounds, residual carbohydrates, minerals, lipids, and hop-derived bioactives, which can be recovered or incorporated into higher-value applications [11,12,13,14]. BSG has attracted particular attention because of its abundance, fibre-rich composition, and suitability for cereal-based food applications [15,16,17,18]. BSY is increasingly investigated as a source of proteins, peptides, β-glucans, mannoproteins, and yeast-derived extracts with technological and nutritional relevance [14,19,20,21]. Spent hops and hot trub are generated in lower amounts, but they may provide phenolic-rich, xanthohumol-rich, antioxidant, flavour-active, or protein-containing fractions for more targeted applications [22,23,24,25].

This transition from waste management to valorization reflects the broader principles of the circular economy. In this context, recovery strategies aim to preserve material value by converting food-processing residues into secondary raw materials and reintegrating them into productive systems. Such approaches can support resource efficiency, cascading use, emission reduction, and the development of new food ingredients or products from underutilized streams [6,8,26,27]. Similar circular-economy strategies have also been reported for other agri-food by-products, including sea buckthorn berry residues incorporated into white chocolate formulations, where product quality and bioactive characteristics were evaluated under a by-product valorization framework [28]. This wider context supports the relevance of brewing by-products as part of a broader shift from disposal-oriented management to food-oriented upcycling.

Scientific interest in brewing by-products has increased considerably during the last decade. Recent studies and reviews have addressed BSG composition and processing, BSY valorization, recovery of bioactive compounds, circular bioeconomy strategies, and the use of brewing residues in food, feed, biotechnology, bio-based products, and bioenergy [6,14,27,29]. However, the literature remains fragmented from the perspective of food ingredient development. Many studies focus on a single by-product, especially BSG, or on a specific valorization route, such as extraction of bioactives, sustainability assessment, or energy recovery. Other contributions adopt a broad circular-economy perspective but provide less detail on food-relevant processing routes, techno-functional performance, sensory quality, safety, regulatory suitability, and scale-up feasibility across different brewing by-products [8,30,31].

This distinction is important because a material with promising composition does not automatically become a viable food ingredient. Food use requires more than the presence of valuable compounds; it also requires stabilization, processability, batch consistency, matrix compatibility, sensory acceptability, safety documentation, regulatory suitability, and industrial feasibility [16,32,33,34]. For example, BSG may be compositionally attractive for fibre enrichment but can negatively affect colour, texture, mouthfeel, and consumer acceptance if particle size and inclusion level are not controlled [16,17,33]. BSY may provide proteins and bioactive cell-wall fractions, but its application depends on controlled lysis, debittering, nucleic acid management, and sensory compatibility [21,35,36]. Similarly, spent hops and hot trub are more suitable for selective recovery of concentrated fractions than for direct bulk incorporation, because bitterness, aroma carry-over, aggregation, and regulatory uncertainty can limit their broader use [22,24,25].

The objective of this review is therefore to provide a critical and translational evaluation of brewing by-products as emerging platforms for food ingredients. Rather than treating these streams only as residues for broad valorization, the review examines them from a food science perspective, with emphasis on ingredient-oriented processing, structure–function relationships, nutritional and techno-functional relevance, safety, sensory performance, regulatory considerations, and industrial translation.

To achieve this objective, the review addresses four specific aims:

(i) To compare the composition, variability, and food-use suitability of the main brewing by-products, namely BSG, BSY, BSH, and hot trub;
(ii) To critically examine stabilization, fractionation, extraction, and bioprocessing strategies that enable their conversion into food-relevant ingredients;
(iii) To assess the nutritional, techno-functional, and sensory implications of incorporating these materials or their recovered fractions into food systems; and
(iv) To identify the main safety, standardization, regulatory, and scale-up barriers that currently limit broader industrial implementation.

The added value of this review lies in its comparative and application-oriented synthesis of brewing by-products as food ingredient platforms. Rather than claiming methodological novelty, the review organizes the available evidence in a form that supports ingredient selection, processing decisions, and identification of short-, medium-, and long-term opportunities for food applications. In contrast to reviews that focus on isolated by-products or broad valorization pathways, the present review addresses the practical question of food translation: which brewing by-products, processing routes, and recovered fractions are closest to realistic use as next-generation food ingredients, and which still require further processing optimization, sensory validation, safety assessment, regulatory clarification, or scale-up studies? By integrating composition, processing, functionality, food applications, safety, and industrial readiness into a unified analytical framework, this review aims to provide researchers, ingredient developers, and food producers with a decision-oriented synthesis of the current state of the field.

## 2. Materials and Methods

This article was designed as a critical narrative review supported by a structured literature search. It was not intended to be a PRISMA-based systematic review, scoping review, or meta-analysis. The review was structured to synthesize and critically evaluate the available literature on brewing by-products as food-relevant ingredient platforms, with emphasis on composition, stabilization, processing, fractionation, techno-functional behaviour, nutritional relevance, sensory quality, safety, regulatory considerations, and industrial translation. The distinction between this approach and a formal systematic review is important because PRISMA-based reviews require a predefined protocol, full record accounting, duplicate removal, exclusion reporting, and a flow diagram, whereas the present review was designed to support a critical and translational synthesis of the literature [37,38].

The literature search covered publications from 2015 to 2026 and was conducted in Web of Science, Scopus, PubMed, and Google Scholar. Web of Science, Scopus, and PubMed were used as the main indexed databases, while Google Scholar was used as a complementary source to identify additional relevant studies, reviews, and cross-disciplinary literature. The final search was conducted in May 2026. The search strategy combined three conceptual blocks: brewing by-products, food-relevant recovery and processing, and food applications or functionality.

The first block included terms related to the main brewing by-products: “brewer’s spent grain”, “brewers spent grain”, “brewer spent grain”, “BSG”, “brewer’s spent yeast”, “brewers spent yeast”, “brewer spent yeast”, “BSY”, “spent hops”, “spent hop”, “hot trub”, “trub”, “brewing by-products”, and “brewery by-products”. The second block included processing and recovery terms such as “valorization”, “valorisation”, “upcycling”, “reuse”, “stabilization”, “stabilisation”, “drying”, “milling”, “fractionation”, “extraction”, “fermentation”, “enzymatic treatment”, “debittering”, “bioprocessing”, and “recovery”. The third block included food application and functionality terms such as “food ingredient”, “food application”, “bakery”, “bread”, “pasta”, “snacks”, “cereal products”, “dairy”, “yogurt”, “yoghurt”, “beverages”, “meat products”, “meat analogues”, “encapsulation”, “techno-functional properties”, “functional properties”, “sensory quality”, “food safety”, and “regulatory”. Database-specific syntax, truncation, and Boolean combinations were adapted according to the requirements of each platform.

Studies were considered eligible when they addressed brewing by-products or fractions derived from them and provided information relevant to food ingredient development, food processing, techno-functional properties, nutritional value, sensory quality, food safety, quality control, regulatory aspects, or industrial implementation. Original research articles were prioritized when evidence was required on processing conditions, product formulation, functionality, safety, sensory response, or product performance. Review articles were used mainly for contextual framing, identification of technological trends, and comparison of broader valorization pathways.

Studies were excluded when they focused exclusively on waste disposal, wastewater treatment, animal feed, bioenergy, composting, non-food biotechnology, or general agro-industrial residues without a specific link to brewing by-products or food-relevant ingredient development. Non-peer-reviewed materials, patents, theses, conference abstracts without sufficient methodological detail, and studies with limited relevance to food applications were also excluded. Studies from feed, biotechnology, bioenergy, or wastewater contexts were considered only when they contained transferable information directly relevant to food-grade stabilization, processing, fractionation, safety, regulatory assessment, or scale-up.

The screening process was performed in two stages. First, titles and abstracts were assessed for relevance to brewing by-products and food-oriented valorization. Second, full texts were evaluated to determine whether they provided information relevant to the objectives of the review. Extracted information included by-product type, processing method, recovered fraction or ingredient form, food matrix or application, reported nutritional or functional effect, sensory limitation, safety issue, regulatory aspect, and scale-up or industrial relevance.

Because the article was conceived as a critical narrative review rather than a systematic review, no formal risk-of-bias assessment, meta-analysis, PRISMA flow diagram, or exhaustive list of excluded studies was prepared. Instead, the extracted evidence was organized thematically to support a comparative and decision-oriented synthesis. Particular attention was given to whether each study provided direct evidence for food applications, proof-of-concept evidence for ingredient functionality, supporting background for processing or safety, or broader contextual information relevant to industrial translation.

Table 1 summarizes the structured literature search framework and eligibility criteria used to assemble the evidence base for this narrative review.

The search framework was designed to support a critical narrative synthesis rather than a formal systematic review. Therefore, the review does not report a PRISMA flow diagram, meta-analysis, formal risk-of-bias assessment, or exhaustive list of excluded studies.

## 3. Brewing By-Products as Food-Relevant Matrices

Brewing generates several residual streams, but their relevance for food ingredient development differs substantially according to their point of generation, composition, stability, structural characteristics, and processing requirements. From a food science perspective, brewer’s spent grain (BSG), brewer’s spent yeast (BSY), spent hops (BSH), and hot trub are the most relevant solid matrices because they contain dietary fibre, proteins, peptides, β-glucans, mannoproteins, phenolic compounds, minerals, bitter acids, lipids, and other constituents that may be recovered or incorporated into food systems [11,14,29,30].

These by-products should not, however, be treated as equivalent food ingredients. Their potential depends not only on the concentration of valuable compounds, but also on moisture content, shelf-life, structural accessibility, sensory profile, safety, batch variability, and compatibility with target food matrices. In this respect, each brewing by-product represents a different ingredient platform and requires a specific stabilization, processing, and application strategy [15,27,31].

As summarized in Table 2, the main brewing by-products originate at different stages of the brewing process. BSG is generated after mashing during wort separation; BSY is recovered mainly after fermentation and clarification; whereas BSH and hot trub originate predominantly during wort boiling, whirlpooling, and clarification. The point of generation strongly influences the composition, perishability, and practical food-ingredient potential of each stream. Therefore, understanding where each by-product is formed is essential for selecting appropriate recovery, stabilization, processing, and food application strategies [5,8,39].

### 3.1. Brewer’s Spent Grain (BSG)

Brewer’s spent grain is the predominant solid by-product of brewing and represents the largest residue stream generated during beer production. It is produced after wort separation and consists mainly of the husk, pericarp, seed coat, and residual endosperm fractions of the original cereal grain. Because of its high availability and its composition rich in dietary fibre and proteins, BSG has received the greatest attention among brewing by-products for direct incorporation into food products and for recovery of food-relevant fractions [11,12,15].

Compositionally, BSG is a lignocellulosic matrix containing high levels of insoluble dietary fibre, mainly cellulose, hemicellulose, arabinoxylans, and lignin, together with moderate to high protein content, residual starch, lipids, minerals, and bound phenolic compounds. These characteristics make BSG relevant for fibre enrichment, protein recovery, phenolic extraction, and the development of cereal-based ingredients with improved nutritional value [13,18,51].

However, BSG also presents important technological limitations. Fresh BSG has high moisture content and is highly susceptible to microbial spoilage, which means that rapid stabilization is required before storage, transport, milling, or incorporation into food systems. In addition, its dark colour, coarse texture, limited dispersibility, and low accessibility of fibre-bound phenolics may restrict its use at high inclusion levels, particularly in products where light colour, fine texture, and neutral flavour are expected [16,17,33].

The composition and functionality of BSG vary substantially according to barley variety, adjunct use, malting conditions, mashing regime, lautering efficiency, drying process, milling degree, and post-production handling. Therefore, BSG should be regarded as a process-dependent substrate rather than a uniform raw material. This variability has direct implications for industrial standardization, formulation consistency, sensory quality, and reproducibility of techno-functional performance in food systems [12,27,31]. As summarized in Table 3, BSG currently appears to be the most realistic brewing by-product for near-term direct food applications, particularly in bakery, pasta, snacks, cereal bars, and other cereal-based products.

### 3.2. Brewer’s Spent Yeast (BSY)

Brewer’s spent yeast is the second major brewing by-product with relevance for food applications. It consists mainly of surplus *Saccharomyces cerevisiae* or *Saccharomyces pastorianus* biomass recovered during fermentation and downstream clarification. In contrast to BSG, which is primarily a fibre-rich cereal matrix, BSY is a cellular matrix containing intracellular nutrients and structurally complex cell-wall components [14,21].

BSY is valuable because of its high protein content, favourable amino acid profile, and presence of β-glucans, mannoproteins, vitamins, minerals, peptides, and other bioactive compounds. These constituents support potential applications in nutritional fortification, flavour enhancement, thickening, emulsification, texture improvement, and encapsulation systems. Yeast-derived β-glucans and mannoproteins are particularly relevant because of their reported techno-functional roles, including water binding, viscosity enhancement, emulsion stabilization, and possible gut-related functionality [20,21,52].

Despite this potential, BSY is less suitable for direct bulk incorporation than BSG. Its valuable components are partly enclosed within a robust cell-wall structure, which limits accessibility unless cell disruption, autolysis, enzymatic hydrolysis, plasmolysis, or mechanical treatment is applied. Moreover, BSY may contain residual hop-derived bitter substances adsorbed onto yeast cell walls, which can negatively affect sensory quality. High nucleic acid content is another important limitation for some food applications and may require reduction before wider use in human food ingredients [35,36].

BSY composition and functional behaviour also vary according to yeast strain, fermentation conditions, repitching cycles, physiological state, recovery point, and beer type. For these reasons, BSY is better positioned as a source of fraction-based ingredients, such as yeast extracts, peptide-rich hydrolysates, β-glucan-rich fractions, and mannoprotein-rich fractions, rather than as a minimally processed bulk ingredient [14,19,21].

### 3.3. Spent Hops (BSH) and Hot Trub

Spent hops and hot trub are generated in smaller quantities than BSG and BSY, but they remain relevant for food ingredient development because they contain concentrated bioactive and functional constituents. Spent hops are mainly produced after wort boiling, whirlpooling, and dry-hopping operations, whereas hot trub is formed during wort boiling and clarification through the aggregation and precipitation of denatured proteins, polyphenols, lipids, malt particles, and hop-derived solids [24,43,44].

BSH is of particular interest as a source of phenolic compounds, bitter acids, prenylflavonoids, xanthohumol, residual proteins, fibres, pectins, and aroma-active compounds. Hot trub is relevant mainly because of its protein-rich and phenolic-containing structure, including protein–polyphenol aggregates and lipid-associated bioactives. These matrices may therefore support the recovery of antioxidant, flavour-active, phenolic-rich, xanthohumol-rich, or protein-containing fractions [23,25,53,54].

However, compared with BSG and BSY, spent hops and hot trub are less appropriate for direct incorporation into food products at high inclusion levels. Their main constraints are strong bitterness, intense flavour carry-over, protein–polyphenol aggregation, low storage stability, and high compositional variability. Their composition and technological behaviour depend on hop variety, hop format, hopping regime, boiling intensity, dry-hopping practice, clarification conditions, and separation efficiency [24,44].

Unless adequate stabilization, debittering, extraction, purification, or fractionation is applied, these constraints may strongly limit food compatibility. Therefore, BSH and hot trub should be considered specialized sources of high-value fractions rather than broadly applicable bulk food ingredients [22,23,55].

### 3.4. Comparative Composition, Variability, and Factors Affecting Ingredient Potential

The comparative suitability of brewing by-products for food applications depends on three connected factors: composition, structural accessibility, and processing feasibility. Table 3 summarizes indicative composition ranges, main food-relevant constituents, variability factors, key constraints, and the most appropriate ingredient directions for BSG, BSY, BSH, and hot trub. The values should be interpreted as approximate literature-based ranges rather than universal specifications, because differences between studies may reflect raw material origin, beer type, brewing conditions, recovery point, stabilization method, and analytical basis.

Taken together, the main brewing by-products differ substantially in composition, processing requirements, and food-ingredient readiness. BSG is the most abundant stream and the most direct candidate for fibre- and protein-enriched food applications. Its use is most realistic in cereal-based products, where moderate incorporation levels can improve nutritional value while maintaining acceptable technological and sensory quality. However, its performance is strongly influenced by moisture control, drying intensity, milling degree, fibre structure, and batch-to-batch variability [15,18,31].

BSY provides a more concentrated source of proteins, peptides, β-glucans, mannoproteins, and soluble bioactive fractions, but its use requires more controlled processing than BSG. The need for cell-wall disruption, bitterness reduction, nucleic acid management, and sensory correction makes BSY more appropriate for fraction-based applications than for direct incorporation of whole biomass [21,35,36].

**Table 3:** Indicative composition ranges, variability factors, and food-ingredient potential of the main brewing by-products.

By-Product	Indicative Composition/Food-Relevant Constituents	Main Variability Factors	Main Food-Relevant Constraints	Most Suitable Ingredient Direction	References
Brewer’s spent grain (BSG)	Fresh BSG is characterized by high moisture, commonly around 70–80% on a wet basis. On a dry basis, it is mainly composed of dietary fibre and carbohydrates, with fibre often representing approximately 40–50% of dry matter. Protein content is commonly reported up to approximately 30%, while lipids are generally lower, often around 7–10%. Important food-relevant constituents include cellulose, hemicellulose, arabinoxylans, lignin, residual starch, minerals, and bound phenolic acids such as ferulic and p-coumaric acids.	Barley variety, adjunct grains, malting conditions, mashing regime, lautering efficiency, drying method, milling degree, and brewery-specific processing conditions.	High moisture, rapid microbial spoilage, short shelf-life, dark colour, coarse texture, gritty mouthfeel, low dispersibility, and limited accessibility of fibre-bound phenolics.	Fibre-rich flours and powders for bakery products, snacks, pasta, cereal bars, breakfast cereals, and other cereal-based foods; protein-, fibre-, arabinoxylan-, or phenolic-rich fractions after pretreatment or extraction.	[11,12,13,15]
Brewer’s spent yeast (BSY)	BSY is a protein-rich biomass, commonly reported to contain approximately 45–60% protein on a dry-weight basis. It also contains peptides, β-glucans, mannoproteins, glycogen, chitin, B vitamins, minerals, antioxidants, and nucleic acids. Yeast cell-wall polysaccharides are especially relevant for thickening, water retention, emulsification, and protective delivery systems.	Yeast species and strain, fermentation type, beer style, repitching cycle, physiological state at recovery, recovery point, and adsorption of hop-derived bitter compounds.	Robust cell-wall structure, limited accessibility of intracellular compounds, residual bitterness, flavour carry-over, high nucleic acid content, and need for controlled cell disruption and debittering.	Yeast extracts, peptide-rich hydrolysates, β-glucan-rich fractions, mannoprotein-rich fractions, savoury flavour ingredients, dairy systems, beverages, meat or hybrid products, and encapsulation systems.	[14,19,20,21,36]
Spent hops (BSH)	BSH contains hop-derived fibres, proteins, bitter acids, phenolic compounds, prenylflavonoids, xanthohumol, pectins, residual lipids, and aroma-active compounds. Protein-containing and xanthohumol-rich fractions can be recovered through selective extraction, although the native matrix is generally less suitable for direct food incorporation.	Hop variety, hop format, hopping regime, boiling intensity, dry-hopping practice, supercritical CO_2_ extraction history, separation efficiency, and storage conditions.	Strong bitterness, intense aroma, flavour carry-over, low bulk suitability, low inclusion tolerance, instability after generation, and need for selective extraction or purification.	Selective recovery of phenolic-rich, xanthohumol-rich, antioxidant, antimicrobial, or flavour-active fractions for low-dose functional applications rather than bulk food use.	[24,25,54,56]
Hot trub	Hot trub consists mainly of protein–polyphenol aggregates, malt particles, hop particles, lipids, carbohydrates, phenolic compounds, minerals, and bitter substances. Reported literature ranges include approximately 40–70% proteins, 20–60% carbohydrates, 1–4% lipids, and 5–10% polyphenols, depending on brewing process and recovery conditions.	Malt type, wort boiling intensity, hop addition, clarification method, beer type, aggregation behaviour, and separation efficiency.	Bitter residues, protein–polyphenol aggregation, instability, low direct applicability, variable composition, browning tendency, and sensory limitations.	Protein-rich and antioxidant fractions after debittering, hydrothermal treatment, purification, or fractionation; limited use in selected pasta, cereal-based, dairy-type, or low-dose antioxidant applications.	[23,43,44,53,55]

**Note:** BSG, brewer’s spent grain; BSY, brewer’s spent yeast; BSH, spent hops. The composition ranges are indicative and should not be interpreted as fixed specifications. Reported values vary according to raw material, brewing process, analytical method, stabilization procedure, and whether results are expressed on a wet or dry basis.

In contrast, BSH and hot trub are generated in lower amounts and have more specialized composition profiles. Their strong bitterness, flavour intensity, instability, and aggregation behaviour make them less suitable for direct bulk use; however, they remain relevant for selective recovery of phenolic-rich, antioxidant, flavour-active, xanthohumol-rich, or protein-containing fractions [22,23,24,25].

Therefore, the suitability of brewing by-products as food ingredients cannot be assessed only by the concentration of valuable compounds. A high content of fibre, protein, phenolics, β-glucans, or mannoproteins is useful only when these constituents can be stabilized, accessed, recovered, and incorporated into food matrices without unacceptable deterioration, cost, safety concerns, or sensory defects. This is especially important for fibre-bound phenolics in BSG, intracellular and cell-wall-associated compounds in BSY, and protein–polyphenol complexes in hot trub [23,35,57].

To avoid overlap between the subsequent sections, the remainder of the review follows a stepwise structure. Section 4 examines the processing operations required to stabilize, modify, and fractionate brewing by-products. Section 5 discusses how these operations influence structure–function relationships and techno-functional behaviour in food systems. Section 6 evaluates product-level food applications, while Section 7 and Section 8 address safety, quality control, standardization, regulatory assessment, scale-up, and industrial translation.

## 4. Processing Strategies Enabling Food Use

**Figure 1:**
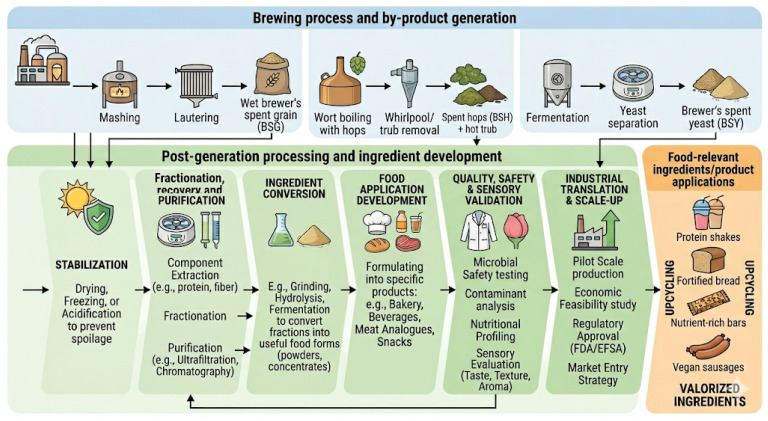
Conceptual workflow linking brewing by-product generation to post-generation processing and food-relevant ingredient development.

After the origin, composition, and ingredient potential of the main brewing by-products have been defined, the next step is to examine the processing operations that make these materials suitable for food use. Table 2 identifies where BSG, BSY, BSH, and hot trub are generated during brewing, whereas Figure 1 illustrates the post-generation sequence required to convert these by-products into food-relevant ingredients and product applications. This distinction is important because by-product generation and ingredient development represent two different stages of valorization.

In this framework, brewing by-products first require stabilization to prevent rapid quality loss. Depending on the target ingredient, this may be followed by milling, fractionation, extraction, purification, enzymatic treatment, fermentation, debittering, or other forms of matrix modification. These operations determine whether a brewing side stream can be transformed into a food-grade ingredient with acceptable stability, functionality, sensory quality, regulatory suitability, and compatibility with the intended food matrix [8,29,58].

Figure 1 provides a conceptual workflow linking brewing by-product generation to post-generation processing and ingredient development. It distinguishes the brewing stages at which wet brewer’s spent grain, spent hops, hot trub, and brewer’s spent yeast are generated from the subsequent processing steps required for stabilization, fraction recovery, ingredient conversion, food application development, quality and safety validation, and industrial translation.

The figure distinguishes the brewing stages at which wet brewer’s spent grain (BSG), spent hops (BSH), hot trub, and brewer’s spent yeast (BSY) are generated from the subsequent processing steps required for stabilization, fractionation, ingredient conversion, food application development, quality, safety and sensory validation, and industrial translation.

### 4.1. Stabilization and Shelf-Life Extension

Rapid stabilization after generation is the first prerequisite for food-grade use of brewing by-products. Fresh BSG, BSY, BSH, and hot trub are highly perishable because they contain substantial moisture and nutrients that can support microbial growth and quality deterioration if processing is delayed. Without timely stabilization, changes in microbiological status, odour, colour, texture, and composition may compromise both safety and subsequent ingredient functionality [9,10,59].

Stabilization has two practical functions. First, it preserves the material long enough to allow collection, transport, storage, and downstream processing. Second, it influences the quality of the final ingredient. For this reason, stabilization should not be regarded as a neutral preliminary operation. Drying, cold storage, freezing, acidification, fermentation, debittering, and physical modification can all affect the sensory profile, functionality, shelf-life, and food-matrix compatibility of the recovered ingredient [8,10,58].

For BSG, drying remains the most common stabilization route when storage, milling, or incorporation into flour-like systems is intended. Because fresh BSG is rich in water and deteriorates rapidly, drying reduces perishability and allows the material to be converted into powders or fractions suitable for bakery, pasta, snack, or cereal-based applications [15,16,17]. However, drying conditions must be selected carefully, because excessive thermal exposure may affect colour, flavour, antioxidant compounds, and functional properties.

For BSY, BSH, and hot trub, stabilization is often linked not only to shelf-life but also to sensory control. BSY may require washing, autolysis control, debittering, or drying to reduce yeast-like flavour and hop-derived bitterness. Spent hops and hot trub may require rapid cooling, drying, debittering, or selective extraction because bitter acids, phenolics, proteins, and lipid-associated compounds can strongly influence flavour and stability [22,24,35,36].

The appropriate stabilization route therefore depends on the intended ingredient form. Bulk cereal-type ingredients, such as BSG flour, require moisture control, microbial stability, and particle-size management. Higher-value fractions, such as protein isolates, phenolic extracts, yeast β-glucans, mannoproteins, or hop-derived compounds, require processing conditions that preserve functionality while controlling bitterness, oxidation, microbial risk, and matrix compatibility.

### 4.2. Fractionation and Extraction Technologies

Once stability has been addressed, fractionation and extraction can be used to obtain ingredients with more defined composition and functionality. These technologies are particularly important when direct incorporation of the raw by-product is limited by fibre rigidity, low solubility, poor dispersibility, bitterness, colour, or the low accessibility of target compounds within the native matrix [11,29,58].

In BSG, fractionation is mainly directed towards the recovery or concentration of protein-rich, fibre-rich, arabinoxylan-rich, and phenolic-rich fractions. Many valuable constituents in BSG are embedded in a lignocellulosic structure, which limits their direct technological use unless pretreatment, milling, hydrolysis, or extraction is applied. Protein extraction, for example, may improve solubility and emulsifying properties, while fibre modification may improve hydration behaviour and compatibility with cereal-based matrices [11,60,61,62].

For BSY, extraction has a different role because many target compounds are intracellular or associated with the yeast cell wall. Cell disruption, autolysis, enzymatic hydrolysis, plasmolysis, ultrasonication, or mechanical treatment may be required before proteins, peptides, β-glucans, mannoproteins, and other functional components can be recovered efficiently. The challenge is to increase accessibility without generating excessive bitterness, undesirable flavour notes, or loss of functional integrity [19,21,35,63].

Spent hops and hot trub are more suitable for selective recovery than for broad direct incorporation. In these matrices, extraction is used to obtain low-dose fractions with specific functions, such as phenolic-rich, xanthohumol-rich, antioxidant, flavour-active, or protein-containing ingredients. However, extraction should not be evaluated only in terms of yield. For food use, the recovered fraction must also show acceptable sensory quality, stability, food-grade processing compatibility, and functionality in the intended matrix [23,24,25,54].

Therefore, the practical value of fractionation depends on the balance between recovery efficiency, ingredient functionality, sensory neutrality, safety, cost, and scalability. A high-yield extract or concentrate is not necessarily useful for food applications if it introduces bitterness, instability, regulatory uncertainty, or poor compatibility with the final product.

### 4.3. Bioprocessing Approaches

Bioprocessing provides an additional route for improving brewing by-products when the objective is not only to recover fractions, but also to modify the matrix itself. Fermentation, enzymatic treatment, autolysis, and controlled hydrolysis can improve digestibility, release bound compounds, modify flavour, increase solubility, and generate ingredients with improved technological or nutritional relevance [35,64,65].

In BSG, bioprocessing is mainly used to loosen the lignocellulosic matrix and increase the accessibility of fibre-associated compounds, proteins, and phenolics. Solid-state fermentation, enzymatic hydrolysis, extrusion-assisted treatments, ultrasound, and pulsed electric field pretreatments may improve phenolic release, protein solubilization, hydration behaviour, and antioxidant activity. However, these effects depend strongly on treatment conditions, microbial strain or enzyme selection, process time, and the intended food application [57,66,67,68].

In BSY, bioprocessing is closely related to controlled cell disruption. Autolysis, enzymatic hydrolysis, plasmolysis, fermentation, and related treatments can release proteins, peptides, β-glucans, mannoproteins, and flavour-active compounds. These processes may improve nutritional and techno-functional value, but they must be carefully controlled because excessive hydrolysis or uncontrolled lysis may increase bitterness, yeast-like notes, or variability in ingredient performance [35,36,69,70].

The strength of bioprocessing is that it can create ingredients that perform differently from the untreated by-product. A fermented BSG base, an enzymatically modified fibre fraction, a peptide-rich BSY hydrolysate, or a yeast-derived β-glucan fraction may offer better compatibility with specific food matrices than the native residue. At the same time, the process must be matched to a defined ingredient objective. Treatments that are useful for bakery products may not be appropriate for beverages, dairy-type systems, meat products, or encapsulation matrices [17,71,72].

### 4.4. Effects of Processing on Nutritional and Functional Performance

Processing determines the nutritional and functional performance of brewing-derived ingredients by changing accessibility, solubility, hydration behaviour, particle size, interfacial properties, sensory load, and stability. The constraints associated with the native matrices are summarized in Table 3; therefore, this section focuses on how processing modifies ingredient behaviour rather than repeating the original limitations of each by-product.

For BSG, processing mainly affects the behaviour of the fibre-rich lignocellulosic matrix. Drying and milling improve storage stability and enable incorporation into flour-like systems, while particle-size reduction influences dispersibility, dough hydration, mouthfeel, and texture. More intensive treatments, such as fermentation, enzymatic hydrolysis, extrusion, ultrasound, or pulsed electric field pretreatment, may increase the accessibility of proteins, phenolics, and carbohydrate fractions. However, overly intensive treatments may negatively affect colour, flavour, cost, or techno-functional performance [17,57,68,72].

For BSY, processing determines the accessibility of intracellular and cell-wall-associated compounds. Autolysis, plasmolysis, enzymatic hydrolysis, mechanical disruption, and ultrasonication can improve the release of proteins, peptides, β-glucans, and mannoproteins. These treatments may support nutritional enrichment, flavour development, emulsification, thickening, or delivery-system applications. However, processing intensity must be controlled so that improved extractability is not accompanied by excessive bitterness, loss of functionality, or poor sensory suitability [19,20,21,35].

For BSH and hot trub, processing is mainly directed towards selective recovery and sensory correction. Extraction, debittering, purification, and fractionation may generate phenolic-rich, xanthohumol-rich, antioxidant, flavour-active, or protein-containing fractions with greater compatibility in food systems. In these cases, the value of processing should be judged not only by recovery yield, but also by the stability, sensory profile, safety, and functionality of the recovered fraction in the target food matrix [22,23,24,55].

Overall, processing routes should be selected according to the intended ingredient role rather than applied as generic valorization steps. A process suitable for producing BSG flour for bakery applications may not be suitable for producing a beverage ingredient, a dairy stabilizer, a savoury yeast extract, or a low-dose hop-derived antioxidant fraction. For food applications, processing success should therefore be assessed through a combined evaluation of stability, composition, functionality, sensory quality, safety, regulatory suitability, and scalability.

## 5. Structure–Function Relationships in Food Systems

The performance of brewing-derived ingredients in food systems depends on the relationship between composition, structure, processing history, and interaction with the target matrix. Brewer’s spent grain (BSG), brewer’s spent yeast (BSY), spent hops (BSH), and hot trub contain proteins, dietary fibre, β-glucans, mannoproteins, peptides, phenolic compounds, bitter acids, minerals, and other food-relevant constituents. However, these compounds do not determine functionality on their own. Their behaviour in foods is strongly influenced by particle size, solubility, hydration capacity, cell-wall resistance, interfacial activity, protein–polyphenol aggregation, bitterness, colour, and compatibility with other food components [11,17,31].

For this reason, the same brewing-derived ingredient may perform well in one product and poorly in another. Fibre-rich BSG fractions may be suitable for bakery, pasta, snack, and cereal-based products, but less compatible with beverages or smooth-textured foods. BSY-derived β-glucans, mannoproteins, peptides, and extracts may provide thickening, stabilization, emulsification, flavour support, or protective delivery functions, but their usefulness depends on cell disruption, fraction purity, bitterness control, and dose. Phenolic-rich extracts from BSG, spent hops, or hot trub may show antioxidant activity at the extract level, but their relevance in real foods depends on stability, sensory threshold, and demonstrated performance in the final matrix [20,21,23,24].

Table 4 summarizes the main structure–function relationships of brewing-derived ingredients in food systems. The table is intended to support ingredient selection by linking ingredient form to functional behaviour, food relevance, and practical limitations.

### 5.1. Water- and Oil-Holding Capacity

Water-holding capacity is one of the most important functional properties of brewing-derived ingredients, particularly those obtained from BSG. This behaviour is mainly associated with the high content of insoluble dietary fibre, including cellulose, hemicellulose, arabinoxylans, and lignin-rich fractions. These components can retain water within the matrix and influence dough hydration, moisture distribution, texture, and mouthfeel in the final product [15,17,31].

In cereal-based formulations, increased water binding may support fibre enrichment and structural modification. However, it can also increase water demand, modify dough handling, reduce loaf volume, or increase product firmness if the formulation is not adjusted. Therefore, water-holding capacity should be treated as a formulation variable rather than as an automatically beneficial property [16,73].

**Table 4:** Structure–function relationships of brewing-derived ingredients in food systems.

Brewing-Derived Ingredient/Fraction	Main Structural or Compositional Feature	Main Functional Behaviour	Main Food Relevance	Practical Limitation	References
Whole or milled BSG flour	High insoluble fibre content; lignocellulosic structure; coarse particles; residual proteins and phenolics	Water binding; texture modification; partial oil binding; fibre enrichment	Bakery products, biscuits, muffins, pasta, cereal bars, snacks, and cereal-based foods	Dark colour, gritty mouthfeel, increased hardness, lower loaf volume, and reduced acceptability at excessive inclusion levels	[15,16,17,18]
Fine or particle-size-controlled BSG fractions	Reduced particle size; larger surface area; improved dispersion in flour-based systems	More uniform incorporation into doughs and batters; modified hydration behaviour	Bakery and cereal-based products requiring smoother texture	Excessive particle-size reduction may alter dough rheology, water absorption, and handling	[31,74,75]
Extruded or physically modified BSG	Thermomechanically modified fibre structure; increased accessibility of soluble components	Improved hydration; modified texture; possible increase in soluble fibre fraction	Snacks, extruded cereal products, bakery products, and functional cereal-based foods	Heat and shear may affect colour, flavour, phenolic stability, and sensory quality	[17,76]
BSG protein-rich fractions	Higher protein concentration; reduced fibre interference compared with whole BSG	Improved solubility, emulsifying activity, and interfacial performance	Protein fortification, emulsified systems, beverages, dairy-type foods, and encapsulation matrices	Functionality depends strongly on extraction route, pH, temperature, denaturation, and drying conditions	[11,60,61,62]
BSG fibre-, arabinoxylan-, or phenolic-rich fractions	Fibre-rich matrix; arabinoxylan-containing hemicellulosic fraction; bound phenolic acids	Water binding; viscosity modification; antioxidant potential after release of bound phenolics	Fibre enrichment, texture support, cereal-based foods, and low-dose functional fractions	Limited accessibility of bound compounds; matrix rigidity; functionality depends on pretreatment	[57,66,77,78]
Whole-BSY biomass	High protein content; β-glucans and mannoproteins within a resistant yeast cell wall; intracellular nutrients	Limited direct functionality unless cell structure is disrupted	Starting material for yeast extracts, protein hydrolysates, β-glucans, and mannoprotein recovery	Residual bitterness, high nucleic acid content, low accessibility of intracellular compounds, and flavour carry-over	[14,21,35]
BSY peptide-rich hydrolysates and yeast extracts	Soluble peptides, amino acids, flavour-active compounds, and released intracellular components	Flavour enhancement; possible antioxidant activity; improved digestibility; savoury notes	Savoury foods, meat products, beverages, hybrid foods, and flavour-supporting systems	Bitterness, yeast-like aftertaste, and dose-dependent sensory limitations	[36,70,79,80]
BSY β-glucan-rich fractions	Yeast cell-wall polysaccharides with water-binding and thickening capacity	Thickening; water retention; texture support; protective carrier effect for probiotic cultures	Fermented dairy or probiotic-related systems, delivery systems, and functional powders	Purity, sensory neutrality, processing reproducibility, and dose control are required	[20,81,82]
BSY mannoprotein-rich fractions	Surface-active yeast cell-wall glycoproteins and mannans	Emulsifying and stabilizing activity; possible viscosity and interfacial effects	Sauces, dairy-type systems, emulsified foods, beverages, and delivery matrices	Controlled autolysis, extraction, and purification are required to preserve functionality	[52,63,83]
Spent hop phenolic- or xanthohumol-rich extracts	Prenylflavonoids, bitter acids, phenolics, and aroma-active compounds	Antioxidant and flavour-related effects at low inclusion levels	Low-dose functional ingredients, flavour-active systems, and specialized extracts	Strong bitterness, aroma carry-over, limited sensory tolerance, and regulatory uncertainty for concentrated extracts	[24,25,54,56]
Hot trub protein-rich fractions	Protein–polyphenol–lipid aggregates; protein-containing and phenolic-associated material	Possible texture contribution, protein enrichment, and antioxidant activity after processing	Selected cereal, pasta, dairy-type, or low-dose functional systems	Bitterness, protein–polyphenol aggregation, low solubility, and variable composition	[22,23,53]
Debittered hot trub fractions	Reduced bitterness after washing or debittering; partial retention of functional compounds	Better sensory compatibility; possible antioxidant or protein contribution	Fresh pasta, processed dairy systems, and selected fortified products	Debittering may also remove soluble nutrients, peptides, minerals, or phenolics	[22,55]
BSG- or BSY-based carrier materials	Fibre-rich, protein-rich, or β-glucan-rich structures capable of forming protective matrices	Encapsulation support; protection of bioactives or probiotic cultures	Functional powders, probiotic carriers, synbiotic systems, and ingredient-delivery systems	Performance depends on carrier composition, co-ingredients, drying method, and storage conditions	[81,84,85,86]

Particle size and processing history strongly influence hydration behaviour. Coarse BSG particles may increase grittiness and reduce structural uniformity, whereas finer fractions may disperse more easily in doughs and batters. At the same time, excessive particle-size reduction can modify rheology and water absorption. This explains why milling degree must be selected according to the target product rather than standardized independently of the food matrix [31,75].

Oil-holding capacity is also relevant in bakery, snack, meat, and hybrid food systems. Fibre-rich BSG fractions and some protein-rich fractions may interact with lipids and modify fat distribution, mouthfeel, and product homogeneity. This can be useful when fat retention contributes to texture, but excessive oil binding may negatively affect sensory quality or processing behaviour. For this reason, hydration and lipid-binding properties should be optimized together with particle size, inclusion level, and matrix composition [87,88,89].

### 5.2. Solubility, Emulsifying, and Foaming Properties

Solubility determines whether brewing-derived fractions can be used in liquid, semi-liquid, or dispersed food systems. Whole BSG has limited solubility because proteins and phenolics remain associated with the lignocellulosic matrix. Therefore, extraction, hydrolysis, air classification, alkaline treatment, or other fractionation approaches are often used to obtain protein-rich fractions with improved solubility and interfacial properties [11,60,61,62].

Emulsifying activity is mainly associated with protein-rich and surface-active fractions. Depending on extraction conditions and degree of denaturation, BSG proteins may contribute to oil–water interfacial stabilization and support texture in emulsified systems. BSY-derived peptides and mannoprotein-rich fractions may also contribute to interfacial behaviour, because yeast mannoproteins contain surface-active structures that can support emulsion stability [21,52,63].

Foaming properties have been less extensively studied than hydration, texture, or emulsification, but they may be relevant in selected food systems where surface activity is required. In general, selectively recovered protein-rich or mannoprotein-rich fractions are more suitable for these applications than whole fibre-rich materials. Functional performance, however, remains dependent on purity, pH, ionic strength, extraction conditions, drying method, and the composition of the final food matrix [11,61,63].

### 5.3. Thickening, Gelling, and Rheological Behaviour

Brewing-derived ingredients can modify viscosity, gel structure, and rheological behaviour, especially when they contain fibre-rich fractions, β-glucans, mannoproteins, proteins, or protein–polyphenol aggregates. BSG often affects texture through its fibre-rich structure and its influence on water distribution. BSY β-glucans and mannoproteins may contribute to thickening, water retention, stabilization, and protective matrix formation [20,52,87].

In bakery and cereal-based products, BSG can increase firmness, modify dough behaviour, and influence product volume. These effects may be useful when the aim is fibre enrichment or structural reinforcement, but they can become negative if they lead to excessive hardness, low volume, poor cohesiveness, or gritty mouthfeel. Processing methods such as milling, extrusion, fermentation, or enzymatic treatment may improve compatibility, but their effects remain matrix-dependent [31,73,74].

Protein-rich fractions from BSG, BSY, or hot trub may also contribute to texture formation in systems where protein interactions are important. However, their functionality depends on solubility, denaturation, aggregation behaviour, pH, ionic conditions, and the presence of phenolics or bitter compounds. In hot trub, protein–polyphenol aggregation may provide antioxidant potential but can also reduce solubility and limit direct use in food systems [23,53].

### 5.4. Antioxidant and Bioactive Functionality

The antioxidant and bioactive potential of brewing-derived ingredients depends on both composition and accessibility. BSG contains phenolic acids, including ferulic and p-coumaric acids, but a substantial part of these compounds is bound to the fibre matrix. As a result, antioxidant activity may increase after treatments that improve release or extractability, such as fermentation, enzymatic treatment, ultrasound, pulsed electric field treatment, or solvent extraction [57,68,77,78].

BSY may provide antioxidant potential through peptides, yeast extracts, and soluble fractions released during autolysis, enzymatic hydrolysis, or cell disruption. However, antioxidant activity measured in an extract or ingredient does not automatically translate into a meaningful effect in a final food product. Dose, stability, sensory contribution, food matrix, processing conditions, and digestion behaviour must be considered before functional relevance can be claimed [21,79,90].

Spent hops and hot trub may contain phenolic-rich, xanthohumol-rich, antioxidant, or protein-associated fractions. These materials are promising for low-dose functional applications, but their use is limited by bitterness, flavour carry-over, poor bulk compatibility, and possible regulatory uncertainty for concentrated fractions. Therefore, antioxidant or bioactive functionality should be described cautiously, distinguishing between in vitro activity, ingredient-level activity, and demonstrated performance in final food matrices [23,24,25,54].

### 5.5. Prebiotic Potential and Digestive Relevance

The digestive relevance of brewing-derived ingredients is mainly associated with dietary fibre, β-glucans, mannoproteins, resistant polysaccharides, and other non-digestible or slowly digestible fractions. BSG provides fibre-rich material that may contribute to gut-related effects, while BSY provides β-glucans and mannoproteins with interest for protective delivery systems and possible microbiota-related applications [13,20,91].

Processing can substantially influence this potential. Fermentation, enzymatic hydrolysis, particle-size reduction, extrusion, and cell-wall disruption may modify the accessibility, solubility, and fermentability of fibre and yeast cell-wall fractions. However, prebiotic or digestive relevance should not be inferred solely from composition. It should be supported by appropriate in vitro digestion, fermentation, microbiota-related, or product-level evidence, depending on the claim being made [81,86,92].

For this reason, the present review treats prebiotic potential as a possible application direction rather than a confirmed property of all brewing-derived ingredients. Stronger evidence is still needed to define dose, matrix effects, stability during processing, sensory acceptability, and actual digestive outcomes in final food products.

### 5.6. Bitterness, Flavour-Active Compounds, and Matrix Interactions

Bitterness, astringency, dark colour, yeast-like flavour, and hop-derived aroma are among the main factors limiting the use of brewing by-products in food systems. In BSG, sensory limitations are often associated with fibre-rich particles, darker colour, phenolic compounds, and changes in texture or mouthfeel. In BSY, residual hop-derived bitter compounds may remain adsorbed onto yeast cell walls, and cell disruption may release flavour-active compounds that are not always compatible with the target food matrix [33,36,70].

BSH and hot trub are particularly affected by bitterness and flavour carry-over because of their association with bitter acids, hop-derived aroma compounds, phenolics, and protein–polyphenol aggregates. These compounds may be valuable at low doses, but they can restrict direct incorporation into products requiring neutral flavour, light colour, or mild aroma. Debittering, washing, deodorization, selective extraction, purification, and dose control are therefore important for improving food compatibility [22,23,55,93].

Matrix interactions are equally important. The behaviour of a brewing-derived ingredient depends not only on its composition, but also on how it interacts with water, starch, gluten, proteins, lipids, polyphenols, minerals, and flavour compounds in the final product. Consequently, the same ingredient may perform differently in bread, pasta, dairy-type systems, beverages, meat products, or encapsulation matrices. This reinforces the need for product-specific formulation trials rather than generalized assumptions about functionality [16,31,87].

Overall, structure–function relationships provide the technical basis for selecting brewing-derived ingredients for food applications. However, functional potential must be interpreted together with processing feasibility, sensory quality, safety, regulatory suitability, and evidence of performance in the intended food matrix. The following section therefore moves from ingredient behaviour to product-level food applications.

## 6. Food Applications of Brewing By-Products

The use of brewing by-products in food systems has progressed from early proof-of-concept studies to formulation-oriented research evaluating nutritional value, technological performance, sensory response, and product feasibility. Whereas Section 5 discussed structure–function relationships at the ingredient level, the present section focuses on product-level applications: where brewing-derived materials have been incorporated, which ingredient forms have been used, and what formulation effects have been reported.

Among the main brewing by-products, BSG remains the most widely studied material for direct food incorporation, mainly because of its high dietary fibre content, moderate protein level, relatively high availability, and compatibility with cereal-based food matrices. BSY, spent hops, and hot trub are less frequently used as bulk ingredients and are more commonly considered as sources of specific fractions, such as yeast extracts, peptides, β-glucans, mannoproteins, phenolic-rich extracts, xanthohumol-rich fractions, or protein-containing materials [14,18,24,31].

Reported inclusion levels and processing conditions vary substantially according to by-product type, pretreatment, particle size, target food matrix, and sensory endpoint. Therefore, this review does not propose universal numerical thresholds for food use. Instead, Table 5 summarizes the main application routes by linking product category, ingredient form, technological or nutritional role, principal formulation effect, limitation, and practical maturity. This approach is intended to distinguish applications that are closer to practical implementation from those that remain dependent on fraction purification, sensory validation, or further pilot-scale testing.

### 6.1. Bakery, Pasta, and Cereal-Based Products

Bakery, pasta, snack, and other cereal-based products remain the most developed food application area for brewing-derived ingredients. In these matrices, BSG has been incorporated mainly as a source of dietary fibre and, to a lesser extent, protein. Bread, biscuits, cookies, muffins, cereal bars, pasta, and extruded cereal products are particularly suitable because their formulations can tolerate moderate levels of fibre-rich ingredients more easily than beverages or smooth-textured foods [18,31,94].

The main nutritional advantage of BSG incorporation is an increase in dietary fibre, often accompanied by partial improvement in protein content or antioxidant-related value, depending on the pretreatment and ingredient form. However, the technological and sensory effects are strongly dose-dependent. Higher BSG levels may increase water absorption, darken the final product, increase firmness, reduce loaf volume, and introduce gritty mouthfeel or cereal-like off-notes [16,17,33].

Processing plays a decisive role in these applications. Milling, particle-size control, fermentation, extrusion, or enzymatic treatment can improve integration into doughs and batters, reduce coarseness, and modify hydration behaviour. Fermented BSG has been used in sourdough bread, while debittered hot trub has been evaluated in fresh pasta as a protein- and antioxidant-containing ingredient [55,73,95,103].

Overall, cereal-based products represent the most realistic near-term route for BSG valorization in human food. This is because the technological function, nutritional message, and consumer understanding of fibre enrichment are relatively clear. Nevertheless, successful formulation still depends on controlling particle size, inclusion level, water balance, colour, and sensory acceptance.

### 6.2. Dairy and Fermented Food Applications

Dairy and fermented systems represent a more selective application area for brewing-derived ingredients. Unlike cereal-based products, these matrices are less tolerant of coarse particles, bitterness, dark colour, or strong flavour carry-over. For this reason, applications in dairy and fermented foods rely more on refined or debittered fractions than on direct incorporation of whole brewing by-products.

BSY-derived β-glucan-rich fractions and yeast-derived protective materials have been investigated for texture support, water retention, and probiotic protection. Their relevance is linked to the ability of yeast cell-wall polysaccharides to form protective or viscosity-modifying structures. However, evidence should be interpreted cautiously because improvements observed in protective systems or model matrices do not automatically translate into broad suitability for yogurt or fermented dairy products [20,81,82].

Hot trub and selected BSG-derived materials have also been explored in dairy-type systems, particularly where antioxidant contribution, protein enrichment, or partial functional improvement is expected. Debittering is essential in such cases, because residual bitterness or aroma carry-over can rapidly reduce acceptability in mild dairy matrices [15,55,113].

Therefore, dairy and fermented food applications should be considered promising but still ingredient-specific. They are more realistic for purified, debittered, or low-dose fractions than for whole wet residues or crude powders. Product-level validation remains necessary to define inclusion level, sensory threshold, texture effects, and compatibility with starter cultures or probiotic microorganisms.

### 6.3. Meat Products, Hybrid Foods, and Meat Analogues

Meat products, hybrid meat systems, and plant-based meat analogues provide another application route for brewing-derived ingredients. In these systems, BSG fractions may contribute fibre enrichment, water retention, fat reduction, or texture modification, while BSY-derived extracts and peptide-rich fractions may support savoury flavour and protein contribution [80,88,89].

The main formulation outcomes are related to moisture retention, texture, matrix homogeneity, and flavour balance. Moderate inclusion levels may support nutritional improvement or technological performance, but excessive addition of fibre-rich or yeast-derived ingredients can increase hardness, visible heterogeneity, bitterness, or aftertaste. Therefore, raw bulk incorporation is generally less suitable than the use of processed fractions with controlled particle size, flavour profile, and functional properties [106,107].

In plant-based meat analogues, BSG can contribute to nutritional improvement and structure formation, but its dark colour, fibre particles, and flavour carry-over may affect product appearance and acceptance. These applications are therefore promising, but they require careful alignment between ingredient properties, extrusion or structuring technology, and the desired sensory profile.

### 6.4. Functional Beverages and Drinkable Systems

Beverages are among the most challenging matrices for brewing-derived ingredients. Compared with solid foods, drinkable systems are less tolerant of turbidity, sedimentation, bitterness, colour changes, coarse particles, and flavour carry-over. Consequently, beverage applications are more realistic for soluble fractions, fermented bases, extracts, or low-dose ingredients than for whole by-products [108,109].

BSG has been considered as a substrate for fermented beverages and as a source of protein or phenolic fractions, while BSY-derived fractions have been studied in relation to fermentation, nutritional enrichment, and sensory improvement. Lactic fermentation of BSY and BSG-derived protein beverage development illustrate that processing can improve compatibility with drinkable systems, but flavour fit remains a major determinant of feasibility [70,108].

Kombucha-like systems and alcohol-free beer models involving non-Saccharomyces yeasts show that brewing-related microorganisms and yeast-derived fractions may contribute to product diversification. However, these applications require careful control of microbial stability, acidity, flavour development, and consumer acceptance [111,112].

For extract-based beverage fortification, phenolic-rich or antioxidant fractions may be attractive at low inclusion levels. Nevertheless, antioxidant activity demonstrated in an extract does not necessarily indicate a meaningful effect in the final beverage. Matrix stability, sensory threshold, bioaccessibility, and regulatory suitability must be demonstrated before such applications can be considered ready for wider use [24,57,77].

### 6.5. Encapsulation and Ingredient-Delivery Systems

Encapsulation and delivery systems represent a more specialized but promising application area for brewing-derived materials. In these systems, the main role of the ingredient is not bulk nutritional enrichment, but protection, stabilization, or delivery of bioactive compounds or probiotic cultures.

BSY β-glucans and spent brewer’s yeast-based carriers have shown potential for protecting probiotic cells or encapsulated compounds during drying, storage, and simulated gastrointestinal exposure. BSG protein- or fibre-based materials have also been explored as carrier systems, including for lipid-based bioactives or probiotic-related applications [81,84,85,86].

The main advantage of this route is that it can use low-inclusion, high-function fractions rather than crude bulk materials. This makes encapsulation particularly relevant for ingredients whose direct incorporation would be limited by texture, flavour, or colour. However, performance depends strongly on carrier composition, co-ingredients, drying method, encapsulation efficiency, storage conditions, and release behaviour.

Although encapsulation is less mature than bakery or cereal-based applications, it may become an important route for higher-value brewing-derived fractions, especially BSY β-glucans, yeast cell-wall materials, BSG proteins, and selected phenolic-rich fractions. Future studies should therefore move beyond carrier preparation and evaluate stability, sensory neutrality, release behaviour, and performance in real food systems.

### 6.6. Overall Assessment of Food Application Readiness

Across product categories, BSG currently has the clearest route towards near-term food use, especially in bakery, pasta, snacks, cereal bars, and other cereal-based products. These applications are supported by a stronger formulation literature, clearer nutritional positioning, and better compatibility with fibre-rich ingredients. The main remaining barriers are sensory quality, particle-size management, colour, texture, and standardization.

BSY is more suitable for medium-term fraction-based applications. Yeast extracts, peptide-rich hydrolysates, β-glucan-rich fractions, and mannoprotein-rich fractions have relevant technological and nutritional potential, but their broader use requires controlled cell disruption, debittering, nucleic acid management, sensory validation, and food-matrix-specific testing.

Spent hops and hot trub are best positioned as specialized sources of low-dose functional fractions rather than as bulk food ingredients. Their potential is linked to phenolic-rich, xanthohumol-rich, antioxidant, flavour-active, or protein-containing fractions, but their practical use remains limited by bitterness, aroma carry-over, instability, purification needs, and regulatory uncertainty. Therefore, while brewing by-products provide several promising food application routes, their readiness differs substantially according to ingredient form, food matrix, processing intensity, and level of product validation.

## 7. Safety and Quality-Control Considerations for Food-Grade Use

The compositional, technological, and sensory constraints of brewing by-products have been discussed in Section 3, Section 4, Section 5 and Section 6. The purpose of this section is therefore not to repeat those limitations, but to define the safety and quality-control requirements that must be met before brewing-derived materials can be considered suitable for food-grade use. This is particularly important because BSG, BSY, BSH, and hot trub are generated within a food-processing environment, but they do not automatically become food-grade ingredients once separated from the brewing process.

For this reason, the section is organized as a hazard-based framework. It addresses microbiological hazards, hygienic recovery, time–temperature control, water activity and moisture control, chemical contaminants, processing-induced risks, allergen and gluten considerations, nucleic acid control in yeast-derived ingredients, sensory validation, ingredient specifications, regulatory documentation, and scale-up validation. This structure is consistent with the logic of general food hygiene, GHP and HACCP principles, which require hazards to be identified, controlled, monitored, verified, and documented throughout the food chain [114,115,116].

Once brewing residues are removed from the controlled brewing stream, they may deteriorate rapidly if collection, storage, transport, or stabilization is delayed. Food-grade valorization therefore requires a documented chain of control from the recovery point to the final ingredient. This includes hygienic handling, defined stabilization conditions, analytical specifications, contaminant screening, traceability, shelf-life validation, and verification that the recovered ingredient is appropriate for its intended food application.

### 7.1. Microbiological Hazards and Hygienic Recovery

Microbiological hazards are particularly relevant for wet brewing by-products because these materials contain moisture and nutrients that may support microbial growth if stabilization is delayed. This concern applies to BSG, BSY, BSH, and hot trub, although the risk profile differs according to the by-product, the recovery point, and the intended food use. The main hazard is not only the microbial status of the material immediately after generation, but also post-separation contamination and growth during collection, storage, transport, and handling.

For BSG, the high moisture content and cereal-derived composition create conditions favourable to rapid spoilage if the material is left unstabilized. For BSY, microbial control must consider both the yeast biomass itself and the risk of contamination during separation, washing, storage, or lysis. For BSH and hot trub, the presence of wet hop particles, coagulated proteins, phenolics, and lipid-associated material may also support quality deterioration unless rapid cooling, drying, or further processing is applied [9,59,117].

Relevant microbiological groups may include total viable aerobic microorganisms, yeasts and moulds, Enterobacteriaceae, coliforms and, depending on the intended food matrix, foodborne pathogens such as *Salmonella* spp., *Listeria monocytogenes*, *Bacillus cereus*, and *Staphylococcus aureus*. Microbiological criteria should not be applied as a single universal threshold for all brewing-derived ingredients. Instead, criteria should be selected according to ingredient form, target food category, intended use level, and shelf-life conditions, following the product-specific approach reflected in microbiological criteria for foodstuffs [118,119].

For food-grade use, the interval between by-product generation and stabilization should be treated as a critical control window. Practical controls include hygienic collection, separation from non-food waste streams, cleaned and closed containers, defined maximum holding times, temperature control where needed, and rapid stabilization by drying, freezing, acidification, fermentation, or downstream processing. Stabilization should be validated rather than assumed, particularly when the ingredient is intended for repeated commercial use.

### 7.2. Water Activity, Moisture Control, and Shelf-Life Validation

Moisture and water activity are central safety and quality parameters for brewing-derived ingredients. High moisture is one of the main reasons why fresh BSG, BSY, BSH, and hot trub have limited shelf-life. In Section 3, moisture was considered as a compositional limitation; in the present section, it is treated as a hazard-control parameter.

For dried BSG, BSY powders, BSH fractions, and hot trub-derived ingredients, both moisture content and water activity should be included in ingredient specifications. A water activity value of approximately 0.85 is commonly used as a practical reference below which many bacterial pathogens are unable to grow, although lower values may be required for stable powders or low-moisture ingredients depending on the target organism, packaging, storage conditions, and intended product category [120]. Therefore, drying should not be evaluated only by final moisture content, but also by water activity, microbiological stability, packaging performance, and shelf-life behaviour.

Shelf-life validation should include microbiological, chemical, functional, and sensory endpoints. For lipid-containing materials, particularly BSG, BSH, and hot trub, oxidation and rancidity should also be considered. For BSY-derived ingredients, uncontrolled autolysis, hydrolysis, or storage may generate off-odours, bitterness, or flavour instability. Consequently, shelf-life testing should be performed on the stabilized ingredient and, where relevant, on the final food matrix.

### 7.3. Chemical Hazards and Contaminant Control

Chemical hazards should be assessed according to raw material origin, brewing inputs, process conditions, recovery point, stabilization method, and downstream processing. For cereal-derived materials such as BSG, relevant hazards include mycotoxins, pesticide residues, heavy metals, and other contaminants associated with barley, malt, adjunct grains, or storage conditions. For spent hops and hop-derived extracts, pesticide residues and hop-associated contaminants may also be relevant. For hot trub, the concentration of protein–polyphenol aggregates, lipids, minerals, and hop-derived solids means that chemical safety should be assessed on the recovered fraction, not only on the original wort or brewing input [23,24,121].

For food-grade use, contaminant control should be aligned with applicable requirements for contaminants and pesticide residues. In the European Union, maximum levels for certain contaminants are established under Regulation (EU) 2023/915, while pesticide-residue requirements are addressed under Regulation (EC) No 396/2005 [122,123]. For BSG and other cereal-derived streams, mycotoxin and heavy-metal screening should be considered, especially when the material is dried, concentrated, or used repeatedly in food production. For hop-derived fractions, pesticide-residue screening should be considered according to raw material origin, cultivation practices, and extraction intensity.

Chemical safety assessment should also consider the effect of concentration and fractionation. Extraction, purification, hydrolysis, drying, or debittering may concentrate desired compounds such as proteins, fibre, phenolics, β-glucans, mannoproteins, or xanthohumol, but the same operations may also concentrate undesirable residues. The final ingredient should therefore be tested according to its intended food use and not evaluated solely on the basis of the original by-product.

### 7.4. Processing-Induced Hazards and Residues from Processing Aids

Processing is necessary to convert brewing by-products into stable and functional ingredients, but it may introduce or concentrate hazards if not properly controlled. Drying, extrusion, roasting, hydrothermal treatment, enzymatic hydrolysis, solvent extraction, debittering, and purification can modify not only functionality and sensory quality, but also chemical safety.

Potential processing-related concerns include heat-induced contaminants, lipid oxidation during drying or storage, concentration of residues during extraction, residual solvents, carry-over of cleaning agents, and residues from enzymes, filtration aids, or other processing aids. For this reason, processing validation should include more than extraction yield or techno-functional improvement. It should also assess food-grade compatibility, residue control, microbiological stability, chemical stability, sensory impact, and shelf-life [114,115].

For phenolic-rich extracts, hop-derived fractions, protein isolates, yeast hydrolysates, and debittered materials, the food-grade status of solvents, enzymes, membranes, filtration aids, adsorbents, and processing aids should be documented. Debittering and washing should also be treated as quality-defining operations because they may reduce undesirable compounds but also remove soluble nutrients, peptides, minerals, or functional phenolics. The objective is therefore not simply to reduce sensory defects, but to preserve an acceptable balance between safety, functionality, composition, and food compatibility.

### 7.5. Allergen and Gluten Considerations

Allergen and gluten issues must be considered before brewing by-products are introduced into human food. BSG originates mainly from barley malt and may therefore contain gluten or gluten-related cereal proteins. This is relevant for labelling, for products intended for gluten-sensitive consumers, and for any claim related to gluten-free status. In the European Union, Regulation (EU) No 1169/2011 establishes food-information requirements and includes cereals containing gluten among substances or products causing allergies or intolerances [124].

BSY, BSH, and hot trub may also contain residues from cereal-derived wort or process streams, depending on recovery point and separation efficiency. Therefore, fractions that are not intrinsically cereal-based should not be considered gluten-free without analytical confirmation. Allergen and gluten management should include raw-material documentation, cross-contact assessment, cleaning validation, ingredient specification, and, where relevant, gluten testing of the recovered ingredient and final food product.

### 7.6. Nucleic Acid Control in Yeast-Derived Ingredients

BSY requires specific safety consideration because yeast biomass may contain substantial levels of nucleic acids, particularly RNA. This is relevant because excessive intake of purine-rich yeast-derived ingredients may contribute to increased uric acid formation, which is important for consumers with hyperuricaemia, gout, kidney disease, or other conditions requiring control of dietary purine intake. For this reason, untreated whole-BSY biomass should be distinguished from processed yeast extracts, protein hydrolysates, β-glucan-rich fractions, and mannoprotein-rich fractions [14,125,126].

For BSY-derived food ingredients, nucleic acid content should be measured and, where necessary, reduced through validated processing. Potential reduction strategies include heat treatment, autolysis, enzymatic processing, plasmolysis, washing, controlled hydrolysis, and fractionation. These processes should not be assumed to be effective without analytical confirmation, because excessive processing may reduce functionality, generate bitterness, or negatively affect sensory quality [35,36,69].

Yeast-derived ingredients should therefore include specifications for nucleic acid content, protein or peptide content, β-glucan or mannoprotein content where relevant, bitterness, microbiological quality, intended use level, and target food category. This documentation is particularly important when BSY fractions are intended for repeated use in savoury products, beverages, meat systems, dairy-type foods, or functional ingredient applications.

### 7.7. Sensory Acceptability as a Quality-Validation Requirement

Sensory limitations such as dark colour, coarse mouthfeel, bitterness, off-notes, and aftertaste are discussed mainly in Section 5 and Section 6 because they relate to structure–function behaviour and product formulation. In the present section, sensory quality is considered as part of the final quality validation. A brewing-derived ingredient may meet microbiological and chemical safety requirements but still be unsuitable for commercial food use if its sensory impact exceeds the acceptable threshold in the target product.

Sensory validation should therefore be integrated into ingredient development rather than treated as a late-stage product adjustment. Sensory evaluation is a key tool in new product development, and acceptance of upcycled or by-product-derived ingredients depends not only on sustainability messages, but also on perceived safety, sensory quality, communication, and trust [34,127,128,129].

Acceptability thresholds are matrix-dependent and should be established experimentally for each ingredient–product combination. For BSG-based ingredients, particle size, colour, fibre structure, and inclusion level are critical. For BSY-derived ingredients, bitterness, yeast-like flavour, hydrolysis-derived off-notes, and nucleic-acid reduction treatments may affect acceptance. For BSH and hot trub fractions, bitter acids, phenolic intensity, aroma carry-over, and protein–polyphenol aggregates may limit use levels. Product-specific sensory testing, dose–response evaluation, and inclusion-level optimization should therefore be included in validation.

### 7.8. Standardization, Traceability, and Ingredient Specifications

Standardization and traceability are essential for repeated food use at industrial scale. Brewing by-products vary according to cereal type, malting conditions, adjunct use, hopping regime, yeast strain, fermentation process, separation point, and post-generation handling. As a result, ingredient performance may vary even when the same by-product category is used. This variability affects safety, functionality, sensory quality, shelf-life, and process reproducibility [9,27].

For food manufacturers, quality consistency requires more than compositional testing. It requires defined sourcing criteria, controlled recovery procedures, validated stabilization, batch characterization, and specifications linked to the intended ingredient function. Relevant specification parameters may include moisture, water activity, particle size, protein, fibre, ash, lipid content, phenolic content, β-glucan or mannoprotein content where relevant, nucleic acid content for yeast-derived ingredients, bitterness indicators, microbiological criteria, contaminant limits, allergen or gluten status, and shelf-life.

Traceability should connect the final ingredient to the brewery source, production date, beer type or process stream, recovery method, stabilization conditions, and subsequent processing steps. This documentation is necessary for quality assurance, regulatory compliance, labelling, recall procedures, and repeated manufacturing. Without such controls, brewing-derived ingredients may remain suitable for laboratory-scale studies but difficult to translate into reliable commercial ingredients.

### 7.9. Regulatory Suitability and Documentation for Food Use

Regulatory suitability must be assessed before commercial use, especially for purified fractions, extracts, hydrolysates, or ingredients obtained through intensive processing. Whole or minimally processed BSG flour may follow a different regulatory pathway from isolated β-glucans, mannoproteins, protein hydrolysates, phenolic-rich extracts, xanthohumol-rich hop fractions, or novel processed ingredients. Legal classification should therefore be evaluated according to ingredient form, processing history, intended use, target food category, use level, and history of consumption.

For purified, concentrated, or newly processed fractions, regulatory screening should consider whether the ingredient form, processing history, intended use level, or history of consumption may require assessment under novel-food requirements. In the European Union, Regulation (EU) 2015/2283 [130] establishes rules for the placing of novel foods on the market and is relevant for ingredients or fractions without a clear history of consumption.

For industrial implementation, safety documentation should include raw-material control, hygienic recovery records, stabilization parameters, processing conditions, analytical specifications, microbiological results, contaminant screening, allergen and gluten assessment, nucleic acid control for BSY-derived ingredients, shelf-life data, sensory validation, labelling considerations, and intended-use justification. These requirements should be addressed early in product development rather than after formulation trials have been completed.

Table 6 summarizes the proposed hazard-based safety and quality-control framework for food-grade brewing-derived ingredients.

Overall, food-grade use of brewing by-products requires more than compositional value or promising techno-functional behaviour. Industrial acceptance depends on whether the ingredient can be recovered hygienically, stabilized rapidly, tested against relevant microbiological and chemical criteria, standardized into reproducible specifications, validated for sensory acceptability, and documented for its intended food use. This hazard-based safety and quality-control framework provides the basis for the industrial translation discussion in Section 8.

## 8. Industrial Translation and Scale-Up Readiness

Industrial translation depends on whether the compositional potential described in Section 3, the processing strategies discussed in Section 4, the structure–function relationships outlined in Section 5, the food applications reviewed in Section 6, and the safety and quality-control requirements summarized in Section 7 can be combined into reproducible, safe, economically feasible, and legally acceptable ingredient routes. For this reason, the present section does not repeat the individual technological, sensory, or safety limitations of each by-product. Instead, it evaluates how these factors influence standardization, regulatory suitability, scale-up readiness, and the practical ranking of brewing-derived ingredients for food use [8,27,29].

### 8.1. Standardization and Specification Setting

For brewing-derived ingredients, industrial implementation requires a transition from by-product recovery to ingredient specification. This means that each material must be described not only by its origin, but also by measurable quality parameters linked to its intended food application.

For BSG-based ingredients, specifications should include moisture, water activity, particle size, fibre content, protein content, colour, microbiological status, gluten/allergen status, and shelf-life. For BSY-derived ingredients, specifications should also include nucleic acid content, bitterness indicators, degree of cell disruption, protein or peptide content, β-glucan or mannoprotein content, and sensory neutrality. For spent hop and hot trub fractions, specifications should focus on phenolic content, protein content where relevant, bitterness, antioxidant activity, residual solvent or processing-aid status, and compatibility with the intended food matrix [9,23,24,27].

Standardization is particularly important because brewing by-products are process-dependent materials. Their composition and performance may vary according to raw material origin, beer type, malting conditions, mashing regime, hopping practice, fermentation conditions, recovery point, and post-separation handling. Therefore, repeated food production requires defined sourcing, controlled recovery, validated stabilization, batch characterization, and certificate-of-analysis parameters linked to the intended ingredient function. Without such controls, promising laboratory results may remain difficult to reproduce under industrial conditions.

### 8.2. Regulatory and Food-Grade Implementation Requirements

Food-grade implementation requires early assessment of the legal status and intended use of each ingredient route. Whole or minimally processed BSG flour may follow a different regulatory pathway from purified β-glucans, mannoproteins, protein hydrolysates, phenolic-rich extracts, xanthohumol-rich hop fractions, or hot trub protein fractions. Regulatory suitability should therefore be assessed according to ingredient form, processing history, target food category, intended use level, history of consumption, and type of claim.

The safety and quality-control requirements discussed in Section 7 provide the basis for this regulatory evaluation. For commercial use, documentation should include raw-material control, hygienic recovery, stabilization records, processing conditions, microbiological criteria, contaminant screening, shelf-life data, compositional specifications, sensory validation, allergen and gluten assessment, labelling considerations, and intended-use justification [115,130].

Regulatory translation differs markedly between whole ingredients and purified or concentrated fractions. In the European Union, regulatory screening should consider general food-law principles, hygiene requirements, food information and allergen labelling, and whether the ingredient may fall within the novel-food framework [130]. In the United States, ingredient use may require assessment under food-additive or GRAS pathways, depending on intended use and available safety evidence [131]. Claims related to “upcycled”, “sustainable”, “clean-label”, “high-fibre”, “source of protein”, antioxidant activity, or functional effects should be substantiated separately from ingredient safety. Such claims should therefore be treated as communication or labelling issues, not as substitutes for safety documentation.

Table 7 compares the main regulatory pathway considerations for brewing-derived food ingredients and supports the industrial readiness assessment presented in the following section.

This comparison shows that regulatory readiness is highest for minimally processed, compositionally characterized ingredients with clearer food-use positioning, such as dried BSG flour. Readiness is lower for purified, concentrated, or bioactive-rich fractions with limited history of consumption. Therefore, the industrial translation of brewing by-products should not be assessed only through processing feasibility or functional performance. It should also include early regulatory screening, intended-use definition, safety documentation, allergen and gluten assessment, labelling review, and claim substantiation.

### 8.3. Industrial Readiness of the Main Ingredient Routes

The reviewed evidence supports a staged interpretation of industrial readiness rather than a single conclusion for all brewing by-products. Dried and milled BSG is the closest route to near-term implementation because it requires relatively simple processing and can be incorporated into food categories that tolerate fibre-rich cereal ingredients, such as bakery products, pasta, snacks, and cereal-based foods. However, commercial use still requires standardized drying, milling, microbiological control, inclusion-level optimization, and sensory validation [18,31,94].

BSY-derived ingredients represent a different route. Yeast extracts, peptide-rich hydrolysates, β-glucans, and mannoproteins are promising for dairy-type products, fermented beverages, savoury foods, meat or hybrid products, and encapsulation systems. Their readiness is more appropriately classified as medium-term because they require controlled cell disruption, nucleic acid reduction, bitterness management, reproducible fractionation, and product-specific sensory validation before wider industrial use [14,21,35,36].

**Table 7:** Regulatory pathway considerations for brewing-derived food ingredients.

Ingredient Type	Processing History	History of Consumption	Intended Use Level	Possible Claim Type	Required Safety Documentation	Likely Regulatory Barrier
Whole dried BSG flour	Drying, milling, particle-size adjustment, possible heat treatment	Stronger history of indirect consumption through cereal-based materials; food use as an upcycled ingredient still depends on product category and jurisdiction	Moderate inclusion in bakery, pasta, snacks, cereal bars, and cereal-based foods	Upcycled ingredient; fibre enrichment; possible source of protein; sustainability-related positioning	Hygienic recovery records; drying and shelf-life validation; microbiological criteria; moisture and water activity; mycotoxin and heavy-metal screening; gluten/allergen status; compositional specification	Allergen/gluten labelling; batch variability; safety documentation for repeated food use; substantiation of upcycled or sustainability claims
Fermented or enzymatically modified BSG ingredients	Stabilization followed by fermentation, enzymatic hydrolysis, extrusion, or other structural modification	Depends on processing intensity and whether the final ingredient differs substantially from conventional cereal-derived materials	Moderate inclusion in bakery, cereal-based foods, beverages, or functional formulations	Fibre enrichment; improved functionality; fermentation-related positioning; clean-label process if applicable	Process description; strain or enzyme documentation; microbiological validation; residual enzyme or processing-aid assessment; compositional and sensory specification	Novel-food screening may be needed if processing significantly changes composition or intended use; process reproducibility and documentation burden
BSG protein-rich or phenolic-rich fractions	Extraction, concentration, purification, drying, possible use of solvents or enzymes	Weaker history of consumption than whole BSG flour because the fraction is concentrated and compositionally different	Low to moderate inclusion depending on protein, antioxidant, or functional role	Protein enrichment; antioxidant potential; functional ingredient; upcycled fraction	Food-grade extraction documentation; residual solvent or processing-aid checks; contaminant screening; protein or phenolic specification; safety and stability data	Higher likelihood of novel-food or food-additive/GRAS assessment; claim substantiation for antioxidant or functional effects
Whole-BSY biomass or yeast extract	Yeast recovery, washing, debittering, autolysis, drying, or extract production	Yeast-derived ingredients have food-use precedents, but brewery-origin biomass requires control of bitterness, contaminants, and nucleic acids	Low to moderate inclusion in savoury foods, beverages, meat products, or flavour systems	Savoury flavour; protein contribution; upcycled yeast ingredient	Hygienic recovery; microbiological testing; nucleic acid specification; bitterness control; contaminant screening; process validation; intended-use level	Nucleic acid/purine-load control; residual bitterness; evidence of safe use at proposed intake; possible GRAS or novel-food assessment depending on form and use
BSY β-glucan-rich fractions	Cell-wall disruption, extraction, purification, drying	More limited history than whole yeast-derived materials because purified β-glucan fractions differ from whole yeast biomass	Low inclusion as texture modifier, water-binding agent, probiotic-protection matrix, or functional fraction	β-glucan-rich ingredient; texture support; possible gut-related or immune-related claims only if legally substantiated	Purity specification; molecular or structural characterization; residual protein/nucleic acid assessment; microbiological and contaminant testing; stability data	Purified fraction may require novel-food or GRAS evaluation; health-related claims require separate authorization or substantiation
BSY mannoprotein-rich fractions	Controlled autolysis, extraction, purification, concentration, drying	Limited direct history as isolated food ingredients compared with whole yeast or yeast extract	Low inclusion for emulsification, stabilization, texture, or delivery systems	Functional stabilizer; emulsifying aid; upcycled yeast-derived fraction	Process documentation; purity and composition; residual nucleic acid or protein profile; microbiological safety; sensory neutrality; stability data	Regulatory classification of purified fraction; limited food-matrix evidence; need to demonstrate safety and technological necessity
Spent hop extracts	Stabilization, extraction, debittering, purification, concentration	Hops have traditional use in beer, but concentrated spent hop extracts used in non-beer foods may have limited history	Very low inclusion due to bitterness and aroma intensity	Natural flavour-related ingredient; antioxidant-rich extract; upcycled hop fraction	Botanical raw-material documentation; pesticide screening; extraction solvent or processing-aid control; bitter-acid and phenolic specification; sensory threshold data	Low sensory tolerance; pesticide and extract-residue control; possible novel-food/GRAS assessment depending on extract composition and use
Xanthohumol-rich spent hop extracts	Selective extraction and concentration of prenylflavonoids	Limited history of consumption as a concentrated isolated fraction	Very low inclusion in specialized functional foods, beverages, or supplements where legally allowed	Xanthohumol-rich fraction; antioxidant or bioactive positioning only with substantiation	Detailed compositional characterization; toxicological and stability data; residual solvent testing; intended intake assessment; claim substantiation	High regulatory scrutiny due to concentrated bioactive; novel-food/GRAS evaluation likely; health claims require separate authorization
Hot trub protein-rich fractions	Recovery, debittering, hydrothermal treatment, protein separation, purification, drying	Limited history as a direct food ingredient; composition depends strongly on wort boiling and clarification	Low to moderate inclusion in selected cereal, dairy-type, or protein-enriched systems	Protein-containing fraction; antioxidant-supporting fraction; upcycled ingredient	Protein and phenolic specification; bitterness control; microbiological testing; contaminant screening; processing-aid residues; shelf-life and sensory validation	Limited consumption history; variable composition; protein–polyphenol aggregation; need for safety and functional validation
Hot trub phenolic or antioxidant fractions	Extraction, purification, concentration, drying	Limited history as concentrated food ingredients	Very low inclusion in targeted antioxidant or functional systems	Phenolic-rich fraction; antioxidant positioning only if substantiated	Phenolic profile; residual solvent or processing-aid testing; contaminant screening; stability data; sensory threshold; intended intake assessment	Novel-food/GRAS assessment may be needed; antioxidant or health-related claims require legal substantiation; bitterness and matrix compatibility

Spent hops and hot trub are less suitable for broad direct incorporation and should be considered specialized ingredient sources. Their most realistic opportunities involve selective recovery of phenolic-rich, xanthohumol-rich, antioxidant, flavour-active, or protein-containing fractions. Because these routes depend on extraction, debittering, purification, matrix compatibility, safety documentation, regulatory assessment, and sensory validation, they are better positioned as long-term or specialized medium-term opportunities rather than immediate bulk food ingredients [23,24,25,54].

Table 8 summarizes the industrial readiness of the main brewing-derived ingredient routes and identifies the main actions required before broader food-sector adoption.

### 8.4. Short-, Medium-, and Long-Term Opportunities

Based on the evidence reviewed, the most realistic near-term opportunity is the use of stabilized and milled BSG in bakery, pasta, snacks, cereal bars, and cereal-based products. These applications are favoured because the processing route is comparatively simple, the by-product is abundant, and cereal matrices can accommodate moderate levels of fibre-rich ingredients. The main barriers are not conceptual feasibility, but standardization, sensory quality, shelf-life, and formulation optimization.

Medium-term opportunities are represented mainly by BSY-derived extracts, peptide-rich hydrolysates, β-glucans, and mannoproteins, as well as by modified BSG fractions. These routes offer higher functional value than crude incorporation, but they require stronger control of processing, sensory quality, safety parameters, and ingredient specifications. Their industrial relevance will depend on whether fractionation and purification can be made reproducible, cost-effective, and compatible with food-grade requirements.

Long-term or specialized opportunities include spent hop extracts, xanthohumol-rich fractions, and hot trub protein or phenolic fractions. These streams are less suitable for direct bulk incorporation, but they may support targeted antioxidant, flavour-active, phenolic-rich, or protein-containing applications at low inclusion levels. Their wider implementation will require additional evidence on sensory thresholds, stability, toxicological safety where relevant, regulatory classification, and performance in final food systems.

Overall, industrial translation should be understood as a stepwise process. The most promising route is not necessarily the one with the highest concentration of bioactive compounds, but the one that combines availability, food-grade processing, reproducible functionality, sensory compatibility, safety documentation, regulatory suitability, and economic feasibility. Under this interpretation, BSG provides the strongest near-term platform, BSY offers medium-term value through controlled fractionation, and spent hops and hot trub remain specialized sources of higher-value fractions that require further validation before broad food-sector adoption.

## 9. Conclusions

This critical narrative review shows that brewing by-products cannot be treated as a uniform group of residues with the same food-ingredient potential. Their suitability for food use depends on the interaction between composition, structural accessibility, stabilization requirements, sensory impact, safety documentation, regulatory status, and industrial feasibility. Among the main brewing by-products evaluated, brewer’s spent grain (BSG) currently represents the most realistic near-term route for food applications, particularly in bakery, pasta, snacks, cereal bars, and other cereal-based products. This is supported by its high availability, relatively simple stabilization route, and composition rich in dietary fibre and proteins. Fresh BSG is characterized by high moisture, commonly around 70–80% on a wet basis, while dry BSG generally contains approximately 40–50% dietary fibre and protein levels that may approach 30%, depending on raw material and processing conditions.

Brewer’s spent yeast (BSY) offers a different but equally relevant opportunity. Its value lies less in direct bulk incorporation and more in controlled fractionation into yeast extracts, peptide-rich hydrolysates, β-glucan-rich fractions, and mannoprotein-rich ingredients. With protein contents commonly reported around 45–60% on a dry-weight basis, BSY is a promising platform for higher-value food applications, including savoury foods, dairy-type systems, fermented beverages, hybrid products, and encapsulation systems. However, its broader use requires controlled cell disruption, debittering, nucleic acid management, sensory validation, and reproducible fractionation.

Spent hops and hot trub appear less suitable for direct incorporation into food products, mainly because of bitterness, aroma carry-over, instability, protein–polyphenol aggregation, and lower tolerance in conventional food matrices. Their most realistic potential lies in selective recovery of low-dose functional fractions, including phenolic-rich, xanthohumol-rich, antioxidant, flavour-active, or protein-containing ingredients. These routes are more specialized and require further extraction optimization, debittering, safety evaluation, regulatory clarification, and product-level validation before they can be considered broadly ready for industrial food use.

The review also shows that compositional richness alone is not sufficient to justify food-grade valorization. A brewing-derived material becomes a viable ingredient only when it can be recovered hygienically, stabilized rapidly, processed reproducibly, incorporated into a compatible food matrix, accepted sensorially, and documented for safety and regulatory compliance. For this reason, the revised framework proposed in this review links by-product origin, composition, processing, structure–function behaviour, food applications, hazard-based safety assessment, regulatory pathways, and industrial readiness.

Overall, the most mature route is the use of dried and milled BSG in cereal-based foods. Medium-term opportunities are represented by modified BSG fractions and BSY-derived extracts, peptides, β-glucans, and mannoproteins. Longer-term or specialized opportunities include spent hop extracts and hot trub-derived protein or phenolic fractions. Future research should therefore move beyond demonstrating compositional potential and focus on standardized recovery, validated stabilization, dose–response formulation trials, sensory thresholds, shelf-life testing, contaminant and allergen control, regulatory assessment, and pilot-scale production. This approach would allow brewing by-products to progress from promising circular-economy resources to reliable, safe, and commercially relevant food ingredients.

## Figures and Tables

**Table 1:** The structured literature search framework and eligibility criteria used for the narrative review.

Methodological Element	Revised Description
Review type	This article was designed as a critical narrative review supported by a structured literature search. It was not designed as a PRISMA-based systematic review, scoping review, or meta-analysis.
Review focus	The review focused on brewing by-products as food-relevant ingredient platforms, with emphasis on composition, stabilization, processing, fractionation, techno-functional behaviour, nutritional relevance, sensory quality, safety, regulatory considerations, and industrial translation.
Databases and sources	Web of Science, Scopus, and PubMed were used as the main indexed databases. Google Scholar was used as a complementary source to identify additional relevant studies, review articles, and cross-disciplinary literature.
Search period	Publications from 2015 to 2025 were considered. The final search was conducted in May 2026.
Main by-product search terms	“brewer’s spent grain”, “brewers spent grain”, “brewer spent grain”, “BSG”, “brewer’s spent yeast”, “brewers spent yeast”, “brewer spent yeast”, “BSY”, “spent hops”, “spent hop”, “hot trub”, “trub”, “brewing by-products”, and “brewery by-products”.
Processing and recovery search terms	“valorization”, “valorisation”, “upcycling”, “reuse”, “stabilization”, “stabilisation”, “drying”, “milling”, “fractionation”, “extraction”, “fermentation”, “enzymatic treatment”, “debittering”, “bioprocessing”, and “recovery”.
Food application and functionality search terms	“food ingredient”, “food application”, “bakery”, “bread”, “pasta”, “snacks”, “cereal products”, “dairy”, “yogurt”, “yoghurt”, “beverages”, “meat products”, “meat analogues”, “encapsulation”, “techno-functional properties”, “functional properties”, “sensory quality”, “food safety”, and “regulatory”.
Boolean search structure	The search combined three conceptual blocks using Boolean operators: brewing by-products AND food-relevant processing or recovery terms AND food application, functionality, safety, or industrial translation terms. Database-specific syntax, truncation, and field codes were adapted according to the requirements of each platform.
Example search string	(“brewer’s spent grain” OR “brewers spent grain” OR “BSG” OR “brewer’s spent yeast” OR “brewers spent yeast” OR “BSY” OR “spent hops” OR “hot trub” OR “trub” OR “brewing by-products” OR “brewery by-products”) AND (“food ingredient” OR “food application” OR bakery OR bread OR pasta OR snacks OR dairy OR beverages OR “meat products” OR “meat analogues” OR encapsulation OR “techno-functional properties” OR sensory OR “food safety” OR regulatory) AND (valorization OR valorisation OR upcycling OR stabilization OR stabilisation OR drying OR milling OR fractionation OR extraction OR fermentation OR “enzymatic treatment” OR debittering OR bioprocessing).
Inclusion criteria	Studies were included when they addressed brewing by-products or fractions derived from them and provided information relevant to food ingredient development, food processing, techno-functional properties, nutritional value, safety, sensory acceptance, quality control, regulatory aspects, or industrial implementation. Original research articles were prioritized for evidence on processing, formulation, functionality, safety, sensory quality, and product performance. Review articles were used mainly for contextual framing and identification of broader technological trends.
Exclusion criteria	Studies were excluded when they focused exclusively on animal feed, bioenergy, wastewater treatment, composting, non-food biotechnology, or general agro-industrial residues without a specific link to brewing by-products and food-relevant ingredient development. Non-peer-reviewed materials, patents, theses, conference abstracts without sufficient methodological detail, and studies with insufficient relevance to food applications were also excluded.
Use of broader search terms	Terms related to feed, biotechnology, bioenergy, and wastewater were not used to broaden the review scope beyond food ingredients. Studies from these areas were considered only when they contained transferable information relevant to food-grade stabilization, processing, fractionation, safety, scale-up, or industrial implementation. Studies focused solely on non-food valorization routes were excluded.
Screening approach	Screening was performed in two stages. First, titles and abstracts were assessed for relevance to brewing by-products and food-oriented valorization. Second, full texts were evaluated to determine whether they provided information relevant to the review objectives.
Extracted information	Extracted information included by-product type, processing method, recovered fraction or ingredient form, food matrix or application, reported nutritional or functional effect, sensory limitation, safety issue, regulatory aspect, and scale-up or industrial relevance.
Evidence classification	The extracted literature was interpreted according to the type of evidence provided: direct evidence for food applications, proof-of-concept evidence for ingredient functionality, supporting background for processing or safety, or broader contextual information relevant to industrial translation.
Methodological limitation	Because the article is a critical narrative review rather than a systematic review, no formal risk-of-bias assessment, meta-analysis, PRISMA flow diagram, or exhaustive list of excluded studies was prepared.

**Table 2:** Simplified brewing process and main points of generation of food-relevant brewing by-products.

Technological Stage	Description of the Stage	Main Resulting By-Product or Relevance to Food-Ingredient Recovery	References
Malting/raw material preparation	Barley is germinated under controlled conditions to activate enzymes required for subsequent starch and protein modification.	No major food-relevant brewing by-product is separated at this stage.	[5,40]
Milling	Malted grains are ground to increase extractability and prepare the grist for mashing.	No major by-product is separated; particle-size control influences wort extraction and later separation efficiency.	[5,40]
Mashing/saccharification	Milled malt is mixed with water, and enzymatic hydrolysis converts starch and part of the protein fraction into soluble compounds.	Insoluble cereal fractions remain in the mash and become the precursor material for BSG.	[40,41]
Wort separation/lautering/filtration	The liquid wort is separated from the insoluble cereal fraction after mashing.	Brewer’s spent grain (BSG), reported at approximately 25 ± 5 kg per 100 L of beer.	[11,15]
Wort boiling with hops	Wort is boiled with hops to develop bitterness, aroma, microbial stability, and colloidal stability. Proteins, polyphenols, and hop-derived solids coagulate during this stage.	Formation of precursors for spent hops and hot trub.	[24,42]
Whirlpool/settling/trub removal	Suspended hop particles, coagulated proteins, polyphenols, and other insoluble materials are separated from the boiled wort.	Spent hops (BSH), approximately 0.3 ± 0.1 kg per 100 L of beer, and hot trub.	[22,43,44]
Cooling and aeration	Clarified wort is cooled and aerated before yeast inoculation.	Mainly process-related effluents; no major solid food-relevant by-product is recovered at this stage.	[39,45]
Fermentation	Yeast converts fermentable sugars mainly into ethanol and carbon dioxide.	Brewer’s spent yeast (BSY), approximately 3 ± 1 kg per 100 L of beer, together with fermentation-related effluents.	[4,14]
Yeast separation/racking	Yeast biomass is separated from fermented beer; part may be reused, while the surplus becomes a by-product.	Brewer’s spent yeast (BSY), approximately 3 ± 1 kg per 100 L of beer.	[14,46]
Maturation/lagering	Beer is conditioned at low temperature to improve flavour stability and clarity.	Small amounts of yeast-rich sediment and process wastewater may be generated.	[39]
Clarification/stabilization/filtration	Residual yeast, suspended particles, and haze-forming compounds are removed.	Additional yeast-rich residues and filtration-related solids may be generated.	[47]
Pasteurization	Beer is heat-treated to improve microbiological stability before distribution.	Mainly process water and effluents; not considered a primary solid food-ingredient matrix in this review.	[48]
Packaging	Beer is filled into bottles, cans, or kegs, with associated washing and rinsing operations.	Brewery wastewater is generated, commonly reported at approximately 3–10 L per 1 L of beer produced; this stream is outside the food-ingredient focus of the present section.	[49,50]

**Table 5:** Food applications of brewing-derived ingredients by product category.

Food Category	Brewing-Derived Ingredient/Fraction	Main Technological or Nutritional Role	Reported or Expected Product Effect	Main Limitation/Formulation Issue	Evidence/Practical Maturity	References
Bread and leavened bakery products	BSG flour, milled BSG, fermented BSG	Fibre enrichment; partial protein increase; modification of dough hydration	Increased dietary fibre content and improved nutritional profile; possible antioxidant contribution depending on pretreatment and inclusion level	Darker crumb, firmer texture, lower loaf volume, and reduced sensory scores at excessive inclusion levels	Near-term application; strongest evidence among food uses	[94,95,96,97]
Biscuits, cookies, crackers, and snack products	BSG flour, BSG-containing flour blends, modified or extruded BSG	Fibre enrichment; texture modification; use as cereal-based ingredient	Improved nutritional profile and suitability for shelf-stable snack products	Increased hardness, gritty mouthfeel, darker colour, and process-dependent acceptability	Near-term application; product-specific optimization required	[98,99,100]
Muffins, cakes, cereal bars, and related bakery products	BSG flour, fine BSG fractions, particle-size-controlled BSG	Fibre enrichment; structure contribution; upcycled product positioning	Increased fibre content and potential consumer interest when nutritional and sustainability benefits are clearly communicated	Particle size and inclusion level strongly influence mouthfeel, texture, colour, and overall liking	Near- to medium-term application; sensory validation needed	[16,18,31,101]
Pasta and cereal-based dough products	BSG flour, treated BSG, BSG-derived ingredients, debittered hot trub	Fibre or protein enrichment; partial replacement of cereal ingredients	Improved nutritional value; possible protein enrichment when suitable BSG- or trub-derived fractions are used	Texture changes, darker colour, rough mouthfeel, and sensory limitations at high inclusion levels	Near- to medium-term application; promising but matrix-dependent	[18,22,102,103,104]
Extruded cereal-based and bakery-type products	BSG flour, extruded BSG fractions, physically modified BSG	Fibre enrichment; structural modification through thermomechanical treatment	Improved fibre profile and modified hydration or texture depending on extrusion conditions	Colour change, flavour carry-over, and need for careful process control	Medium-term application; processing-dependent feasibility	[31,73,74,76]
Fermented dairy and probiotic-related systems	BSY β-glucan-rich fractions; yeast-derived protective materials	Water retention; texture support; possible probiotic protection	Potential texture-supporting or protective role for probiotic cultures, depending on matrix, dose, and processing conditions	Dose-dependent aftertaste, flavour carry-over, purity requirements, and need for product-specific validation	Proof-of-concept to medium-term; stronger validation needed in final products	[22,81,82]
Processed cheese and dairy-based systems	Debittered trub fractions; selected BSG- or BSY-derived functional fractions	Antioxidant contribution; partial functional enrichment; possible texture contribution	Improved functional enrichment or oxidative stability in selected dairy matrices, depending on fraction type	Bitterness, flavour compatibility, and matrix-specific validation must be controlled	Proof-of-concept to medium-term; ingredient-specific	[36,55,105]
Meat products	BSY extracts; selected BSG fibre/protein fractions where matrix-compatible	Savoury flavour support; water-binding or texture support; partial nutritional enrichment	Possible flavour improvement and formulation support depending on ingredient type and use level	Excess fibre or yeast-derived flavour may increase hardness, heterogeneity, or aftertaste	Medium-term; feasible when dose and sensory profile are controlled	[80,89,106]
Hybrid meat products and meat analogues	BSG fractions; BSY extracts and peptide-rich fractions	Texture support; protein/fibre enrichment; savoury flavour contribution	Improved nutritional profile and possible flavour support in hybrid or plant-based matrices	Colour, flavour carry-over, visible heterogeneity, and matrix uniformity remain limiting factors	Medium-term; growing but still formulation-dependent	[88,89,107]
Functional beverages	BSG-derived fermented bases; BSG protein or phenolic fractions; BSY fractions	Antioxidant enrichment; amino acid contribution; fermentation or functional support	Potential increase in antioxidant activity, nutritional value, or fermentation-related functionality	Turbidity, bitterness, aftertaste, sedimentation, and poor compatibility of coarse particles	Medium-term; soluble fractions more realistic than bulk material	[70,108,109,110]
Fermented plant-based drinks and kombucha-like systems	BSY extracts; yeast-derived fractions; selected non-*Saccharomyces* yeasts	Fermentation support; functional enrichment; possible probiotic-related role	Added biological activity or stability support depending on beverage matrix	Strong dependence on flavour fit, visual acceptability, microbial control, and consumer acceptance	Proof-of-concept to medium-term	[70,111,112]
Beverages with extract-based fortification	Phenolic-rich extracts from BSG, spent hops, spent yeast, or hot trub	Low-dose antioxidant or bioactive addition	Potential use of targeted fractions without incorporating bulk solids	Final effect depends on extract stability, beverage matrix, flavour compatibility, and sensory threshold	Proof-of-concept; requires validation in final beverages	[24,56,57,77]
Encapsulation and delivery systems	BSY β-glucans; spent brewer’s yeast carrier materials; BSG protein or fibre-based carriers	Carrier function; probiotic protection; delivery of bioactive compounds	Improved protection of probiotic cultures or encapsulated bioactives during processing, storage, or simulated digestion	Performance depends on carrier design, co-ingredients, drying method, and storage conditions	Specialized medium-term application; promising for high-value fractions	[81,84,85,86]
Low-dose functional ingredient systems	Spent hop extracts; hot trub protein- or phenolic-rich fractions	Antioxidant, flavour-related, or partial fortification role	Use of high-activity fractions at low inclusion levels	Bitterness, aroma carry-over, matrix compatibility, regulatory suitability, and claim substantiation may limit broader use	Longer-term or specialized use; requires stronger safety and sensory validation	[23,24,25,53]

**Note:** This table reports food applications and potential application routes only where the cited literature supports the relevant food matrix, ingredient type, or functional effect. Where the available evidence is based on extraction, fraction characterization, in vitro activity, or proof-of-concept rather than direct product formulation, the wording is intentionally conservative.

**Table 6:** Hazard-based safety and quality-control framework for brewing-derived food ingredients.

Hazard/Control Area	Main Affected By-Product(s)	Food-Grade Concern	Suggested Control or Assay	Mitigation/Validation Approach	References
Hygienic recovery	BSG, BSY, BSH, hot trub	Contamination during separation, collection, transfer, or storage	Defined recovery point; sanitation records; batch identity	Hygienic collection; cleaned containers; controlled transfer; traceability from brewery stream to ingredient batch	[114,115,116]
Time–temperature control	BSG, BSY, BSH, hot trub	Microbial growth and quality loss before stabilization	Holding time; storage temperature; time to drying, freezing, acidification, or processing	Maximum holding time; cold storage where needed; immediate stabilization	[9,59]
Microbiological hazards	BSG, BSY, BSH, hot trub	Spoilage and possible foodborne hazards in unstabilized wet residues	Total viable count; yeasts and moulds; Enterobacteriaceae; coliforms; *Salmonella* spp.; *Bacillus cereus*; *Bacillus cereus*; *Staphylococcus aureus* where relevant	Validated stabilization; product-specific microbiological criteria; shelf-life testing; hygiene monitoring	[59,117,119]
Water activity and moisture	Dried BSG, BSY powders, BSH, hot trub fractions	Residual water may permit microbial growth or quality deterioration	Moisture content; water activity; packaging integrity	Drying validation; water activity specification; packaging and storage validation	[9,130]
Mycotoxins	Mainly BSG; possibly cereal-derived trub fractions	Carry-over from barley, malt, adjunct grains, or storage	Mycotoxin screening according to cereal risk profile	Supplier control; raw-material screening; high-risk batch testing; rejection criteria	[121,122]
Heavy metals	BSG, BSH, hot trub, concentrated fractions	Carry-over from raw materials or concentration during fractionation	Lead, cadmium, arsenic, mercury, and nickel where relevant	Supplier declarations; analytical testing; compliance with applicable maximum levels	[121,122]
Pesticide residues	BSG, BSH, hop-derived extracts, hot trub	Residues from barley, adjunct grains, or hops may persist or concentrate	Targeted pesticide-residue screening according to raw material and regional MRL requirements	Supplier control; residue analysis; compliance verification before food use	[118,121]
Cleaning agents and processing-aid residues	Extracts, hydrolysates, debittered materials, purified fractions	Carry-over from equipment cleaning, solvents, enzymes, adsorbents, or filtration aids	Processing-aid documentation; residual solvent checks where applicable; cleaning validation	Food-grade processing routes; validated rinsing and cleaning; supplier documentation	[114,116]
Heat-induced or processing-related contaminants	Heat-treated BSG, extruded materials, roasted or dried fractions	Excessive thermal load may create undesirable compounds or reduce quality	Process temperature and time monitoring; product-specific contaminant checks where relevant	Optimized drying or extrusion; validation of thermal process	[114]
Lipid oxidation and rancidity	BSG, BSH, hot trub, lipid-containing fractions	Storage-dependent rancidity and sensory deterioration	Peroxide value; TBARS; free fatty acids; sensory screening where relevant	Oxygen and light control; adequate packaging; shelf-life testing	[9,121]
Protein–polyphenol aggregation	Hot trub and some BSH-derived fractions	Reduced solubility, difficult purification, and limited direct use	Protein solubility; phenolic content; turbidity; functional testing	Controlled extraction; selective fractionation; matrix-specific functionality testing	[23,24]
Bitterness and off-notes	BSY, BSH, hot trub	Reduced acceptability and inconsistent product quality	Sensory threshold testing; bitterness indicators; aroma screening	Washing; debittering; deodorization; dose control; matrix selection	[34,127,129]
Gluten and cereal allergens	Mainly BSG; also BSY, BSH, hot trub if wort-derived residues remain	Barley-derived gluten may affect labelling, consumer suitability, and gluten-free claims	Gluten testing where relevant; allergen risk assessment; raw-material documentation	Allergen management; cross-contact control; label compliance; no gluten-free claim without analytical confirmation	[124]
Nucleic acid control	BSY and yeast-derived fractions	High RNA or purine load may restrict direct use of untreated yeast biomass	RNA or total nucleic acid content; intended use level; target consumer group	Heat treatment, autolysis, enzymatic hydrolysis, washing, fractionation; validation of nucleic-acid reduction	[69,125,126]
Ingredient specification	All brewing-derived ingredients	Variable composition and inconsistent food performance	Moisture, water activity, particle size, protein, fibre, ash, lipids, phenolics, β-glucans, mannoproteins, and nucleic acids where relevant	Certificate of analysis; batch-release criteria; validated specifications linked to intended food use	[9,27]
Shelf-life validation	All stabilized ingredients	Loss of safety, functionality, or sensory quality during storage	Microbiological stability; water activity; oxidation indicators; sensory profile; functional properties	Accelerated and real-time shelf-life studies; packaging validation; storage instructions	[114]
Sensory validation	All ingredients intended for direct food use	Ingredient may be safe but unacceptable in the target food matrix	Trained-panel or consumer testing; dose–response assessment; inclusion-level optimization	Matrix-specific sensory validation; recommended use levels; product-category guidance	[34,127,128,129]
Regulatory suitability	Whole ingredients, purified fractions, hydrolysates, extracts	Unclear legal status for concentrated or novel fractions	Food-law screening; history of consumption; ingredient form and use level	Regulatory assessment; safety dossier where needed; labelling and use-level justification	[115,131]
Scale-up validation	All brewing-derived ingredients	Laboratory safety and functionality may not transfer to repeated industrial production	Repeated-batch testing; pilot-scale trials; process capability data	Pilot production; batch-to-batch comparison; HACCP plan; process control plan	[27,114]

**Note:** This table provides a hazard-based framework rather than fixed universal limits. Specific microbiological criteria, contaminant limits, water activity targets, allergen requirements, regulatory documentation, and validation procedures should be selected according to ingredient form, target food category, intended use level, jurisdiction, and validated shelf-life.

**Table 8:** Industrial readiness matrix for brewing-derived food ingredient routes.

Ingredient Route	Readiness Category	Current Basis for Classification	Main Industrial Barrier	Priority Next Steps
Dried and milled BSG flour for bakery, pasta, snacks, and cereal-based products	Near-term	High availability; relatively simple stabilization and milling; compatibility with cereal-based matrices; multiple formulation studies	Moisture control, microbial stability, particle-size standardization, colour and texture impact, sensory limits at high inclusion levels	Standardized drying and milling protocols; microbiological and shelf-life validation; inclusion-level optimization; batch-to-batch specifications
Fermented or enzymatically modified BSG ingredients	Medium-term	Improved nutritional and functional potential compared with untreated BSG; relevance for bakery, cereal, and beverage-related applications	Process reproducibility, enzyme or strain selection, cost, variable sensory effects, and limited pilot-scale evidence	Pilot-scale fermentation or enzymatic trials; process validation; sensory testing; clear ingredient specifications
BSG protein-rich or phenolic-rich fractions	Medium-term	Higher-value fractions with potential use in fortified, antioxidant, or functional food systems	Extraction cost, food-grade processing requirements, yield–functionality balance, colour, and flavour effects	Food-grade extraction optimization; functionality testing in target matrices; stability and safety assessment
BSY extracts and peptide-rich hydrolysates	Medium-term	Strong nutritional and flavour potential; relevance for savoury foods, beverages, meat products, and hybrid products	Residual bitterness, nucleic acid content, flavour carry-over, and need for controlled lysis	Debittering and nucleic acid reduction; sensory thresholds; compositional specifications; repeated-batch validation
BSY β-glucan- and mannoprotein-rich fractions	Medium-term	Clear potential for thickening, stabilization, emulsification, texture support, and probiotic protection	Purification cost, sensory neutrality, reproducibility, and regulatory positioning of purified fractions	Purity and functionality standards; food-matrix trials; scale-up testing; regulatory screening
Spent hop phenolic- or xanthohumol-rich extracts	Long-term/specialized medium-term	High-value bioactive potential, but more suitable for low-dose applications than bulk food use	Strong bitterness, aroma carry-over, low inclusion tolerance, extraction cost, and regulatory uncertainty	Selective extraction and debittering; dose–response sensory studies; stability testing; safety and legal assessment
Hot trub protein-rich or antioxidant fractions	Long-term/specialized medium-term	Protein and antioxidant potential reported, but direct food use remains limited	Protein–polyphenol aggregation, bitterness, instability, purification needs, and limited sensory compatibility	Debittering and fractionation; functionality testing in selected matrices; pilot-scale recovery; safety and shelf-life validation

## Data Availability

No new data were created or analyzed in this study.
